# Trajectories of prescription opioid dose and risk of opioid-related adverse events among older Medicare beneficiaries in the United States: A nested case–control study

**DOI:** 10.1371/journal.pmed.1003947

**Published:** 2022-03-15

**Authors:** Yu-Jung Jenny Wei, Cheng Chen, Motomori O. Lewis, Siegfried O. Schmidt, Almut G. Winterstein

**Affiliations:** 1 Department of Pharmaceutical Outcomes and Policy, University of Florida College of Pharmacy, Gainesville, Florida, United States of America; 2 Center for Drug Evaluation and Safety, University of Florida, Gainesville, Florida, United States of America; 3 Department of Community Health and Family Medicine, University of Florida College of Medicine, Gainesville, Florida, United States of America; 4 Department of Epidemiology, University of Florida Colleges of Medicine and Public Health and Health Professions, Gainesville, Florida, United States of America; Massachusetts General Hospital, UNITED STATES

## Abstract

**Background:**

Despite the rising number of older adults with medical encounters for opioid misuse, dependence, and poisoning, little is known about patterns of prescription opioid dose and their association with risk for opioid-related adverse events (ORAEs) in older patients. The study aims to compare trajectories of prescribed opioid doses in 6 months preceding an incident ORAE for cases and a matched control group of older patients with chronic noncancer pain (CNCP).

**Methods and findings:**

We conducted a nested case–control study within a cohort of older (≥65 years) patients diagnosed with CNCP who were new users of prescription opioids, assembled using a 5% national random sample of Medicare beneficiaries from 2011 to 2018. From the cohort with a mean follow-up of 2.3 years, we identified 3,103 incident ORAE cases with ≥1 opioid prescription in 6 months preceding the event, and 3,103 controls matched on sex, age, and time since opioid initiation. Key exposure was trajectories of prescribed opioid morphine milligram equivalent (MME) daily dosage over 6 months before the incident ORAE or matched controls. Among the cases and controls, 2,192 (70.6%) were women, and the mean (SD) age was 77.1 (7.1) years. Four prescribed opioid trajectories before the incident ORAE diagnosis or matched date emerged: gradual dose discontinuation (from ≤3 to 0 daily MME, 1,456 [23.5%]), gradual dose increase (from 0 to >3 daily MME, 1,878 [30.3%]), consistent low dose (between 3 and 5 daily MME, 1,510 [24.3%]), and consistent moderate dose (>20 daily MME, 1,362 [22.0%]). Few older patients (<5%) were prescribed a mean daily dose of ≥90 daily MME during 6 months before diagnosis or matched date. Patients with gradual dose discontinuation versus those with a consistent low dose, moderate dose, and increase dose were more likely to be younger (65 to 74 years), Midwest US residents, and receiving no low-income subsidy. Compared to patients with gradual dose discontinuation, those with gradual dose increase (adjusted odds ratio [aOR] = 3.4; 95% confidence interval (CI) 2.8 to 4.0; *P* < 0.001), consistent low dose (aOR = 3.8; 95% CI 3.2 to 4.6; *P* < 0.001), and consistent moderate dose (aOR = 8.5; 95% CI 6.8 to 10.7; *P* < 0.001) had a higher risk of ORAE, after adjustment for covariates. Our main findings remained robust in the sensitivity analysis using a cohort study with inverse probability of treatment weighting analyses. Major limitations include the limited generalizability of the study findings and lack of information on illicit opioid use, which prevents understanding the clinical dose threshold level that increases the risk of ORAE in older adults.

**Conclusions:**

In this sample of older patients who are Medicare beneficiaries, 4 prescription opioid dose trajectories were identified, with most prescribed doses below 90 daily MME within 6 months before ORAE or matched date. An increased risk for ORAE was observed among older patients with a gradual increase in dose or among those with a consistent low-to-moderate dose of prescribed opioids when compared to patients with opioid dose discontinuation. Whether older patients are susceptible to low opioid doses warrants further investigations.

## Abstract 2

Yu-Jung Jenny Wei and colleagues study patterns of prescribed opioid doses in the 6 months before an opioid-related adverse event among Medicare beneficiaries with chronic non-cancer pain in the US.

## Author summary

**Why was this study done?:**

- The number of older adults with medical encounters for opioid-related adverse events (ORAEs) has increased over the past decade.
- Yet, little is known about patterns of prescription opioid dose and their association with risk for ORAEs among older patients.

**What did the researchers do and find?:**

- We assessed trajectories of opioid morphine milligram equivalent (MME) dose prescribed during the 6 months before an incident ORAE for 3,103 cases and during the 6 months before the equivalent date for 3,103 controls who did not experience an ORAE, selected from a cohort of Medicare older patients diagnosed with chronic noncancer pain who were new users of prescription opioids.
- We found four prescription opioid dose trajectories, with most prescribed doses below 90 daily MME before ORAE or matched date.
- We also found that compared to patients with gradual dose discontinuation (from ≤3 to 0 daily MME), those with gradual dose increase (from 0 to >3 daily MME), consistent low dose (between 3 and 5 daily MME), and consistent moderate low (>20 daily MME) had a higher risk for ORAEs.

**What do these findings mean?:**

- An increased risk for ORAE was observed among older patients who had a gradual increase in dose or among those who had a consistent low-to-moderate dose of prescribed opioids when compared to patients with opioid dose discontinuation.
- Whether older patients are susceptible to low opioid doses warrants further investigations.

## Introduction

The number of older adults who had medical encounters for treatment of opioid misuse, dependence, and poisoning has increased disproportionately over the past decade [1]. The opioid-related adverse events (ORAEs) defined by the United States (US) government agencies [1–3] contain diagnostic codes commonly used for opioid use disorder (OUD) and overdose from use of illicit opioids (i.e., heroin) or incorrect use of prescribed opioids, as well as E codes for severe adverse effects from use of heroin or correct use of prescribed opioids that lead to hospital or emergency department visits. The rate of hospital stays and emergency department visits due to ORAEs rose by 34% (from 199.3 stays to 267.6 stays per 100,000 persons) and 74% (from 44.7 visits to 77.9 visits per 100,000 persons), respectively, among older patients between 2010 and 2015 [1]. A study of a commercially insured population also indicated a marked increase (14.2-fold from 2.05 to 31.12 per 10,000 persons) in the incidence of OUD or overdose among older adults aged 65 and older between 2006 and 2016 [4]. These alarming statistics have prompted questions of what might have predisposed older patients to be at risk for ORAEs [2].

Of the known risk factors, prescription opioid dose is one of the strong predictors of ORAEs [5–9]. Studies of nonelderly or mixed populations of young and older populations showed that use of prescription opioids at a dose of 90 morphine milligram equivalent (MME) or above per day was associated with an increased risk for opioid overdose and deaths [10–13]. Built on this evidence, the 2016 Centers for Disease Control and Prevention (CDC)’s Guidance for Opioid Prescribing for Chronic Pain recommends avoidance of prescribing daily opioid doses at 90 mg MME or greater [14]. Since then, some medical societies’ guidelines [15], state regulations [16], and health insurance payers [17] have adapted the CDC-recommended dose threshold and limited prescribing doses of opioids to 90 mg MME per day [18,19].

The Centers for Medicare and Medicaid Services (CMS), the largest insurer of older adults in the US, has utilized 90 daily MME as one of the criteria for flagging high-risk beneficiaries for OUD or overdose and required its Part D plan sponsors to adjudicate the appropriateness of opioid prescribing of these high-risk patients [20]. However, our prior study has shown that the CMS’s opioid overutilization criteria missed the majority of patients with OUD or overdose and flagged more than half of opioid prescription users as high risk who were not diagnosed with OUD or overdose [21]. The finding challenges the use of 90 daily MME as a risky threshold for Medicare beneficiaries, the vast majority of whom are aged 65 years or older. Literature has primarily focused on establishing the high-risk prescription opioid dose thresholds using healthcare data among young (aged 18 to 64) individuals, which may not be applicable to older individuals who may have different thresholds for adverse opioid outcomes due to declined renal and hepatic function, multiple comorbidities, and polypharmacy.

Because of the time-varying nature of prescription opioid use, assessing the progression of opioid dose toward ORAEs is important to understand whether there are typical and atypical opioid dose patterns emerged before the adverse events. Thus, the present study aims to (1) examine trajectories of prescription opioid dose preceding the incident medical encounter for ORAEs; and (2) quantify the association between identified trajectories of prescribed opioid dose and risk of ORAEs among Medicare older adults with chronic noncancer pain (CNCP).

## Methods

### Study setting and cohort

Using the pharmacy and medical claims data from a 5% national random sample of Medicare beneficiaries from the US CMS [22], we conducted a nested case–control study design within a cohort of older (≥65 years) beneficiaries enrolled in US Medicare who were new opioid users and had a diagnosis of CNCP between January 1, 2011 and December 31, 2018. We chose a nested case–control study design because such design allows for examination of prescription opioid use in a time window (i.e., 6 months in this study) preceding ORAE outcome as a risk factor [23]. Studying prescription opioid use before ORAE is important because of the time-varying nature of opioid use, with a larger effect expected from opioid exposure preceding an ORAE compared to distant opioid exposure during the early months of opioid initiation.

Cohort members were older adults aged 65 or older and naïve to opioids for 12 months prior to the date of their first dispensed opioid prescription (i.e., cohort entry). During the 12-month pre-cohort entry, patients were also required to have the following: (1) continuous enrollment in Medicare Parts A (inpatient), B (outpatient provider), and D (prescription drug) without insurance coverage from Health Maintenance Organization or employer-sponsored plans; and (2) primary or secondary diagnosis of a chronic pain condition (**S1 Table**) to ensure a relatively homogeneous cohort regarding pain conditions. We excluded patients who received a cancer diagnosis, hospice care, or palliative care, as well as those with a history of an ORAE encounter during the year before cohort entry. Patients were followed until an ORAE event, a cancer diagnosis, receiving palliative or hospice care, death, Medicare disenrollment, or study end (i.e., December 31, 2018), whichever came first. The University of Florida Institutional Review Board approved the study with a waiver of informed consent and HIPAA authorization because of minimal risk and lack of feasibility to contact Medicare patients. Data analyses were performed as per a prespecified protocol between January and December 2020 (**S1 Text**). This study followed the Strengthening the Reporting of Observational Studies in Epidemiology (STROBE) reporting guidelines (**S1 STROBE Checklist**).

### Selection of cases and controls

In the cohort of opioid initiators, we identified cases of ORAE using *the International Classification of Diseases*, *Ninth or Tenth Revision*, *Clinical Modification (ICD-9 or ICD-10 CM) Codes* recorded in inpatient or outpatient encounter claims during follow-up. These codes have been used by the CDC and Agency for Healthcare Research and Quality (AHRQ) to define ORAEs, including opioid misuse (ICD-9 codes: 305.50–305.52), opioid dependence and unspecified use (304.00–304.02, 304.70–304.72), opioid poisoning (965.00–965.02, 965.09, 970.1, E850.0-E850.2), and adverse effects of opioids (E-codes: E935.0-E935.2, E940.1) [1,3]. We also used the ICD-9-CM to ICD-10-CM code conversion of ORAEs provided by AHRQ (**S1 Table**) [24]. Opioid misuse and dependence are commonly grouped as OUD, and opioid poisoning is also known as OD. The adverse effects of opioids defined by the E codes include any severe reactions to illicit opioids (i.e., heroin) or correct use of prescribed opioids (i.e., methadone, opioid antagonists, and other opioids) that lead to an emergency department or hospital visit. Consistent with prior studies [4,25], when identifying patients with incident ORAE encounter, we excluded ICD-10-CM codes that indicated “in remission” or “subsequent encounter.” The date of the first ORAE encounter represented the “index” date.

To emulate clinical practices where a limited time window of patient history is often available for routine clinical assessment, we focused on trajectories of opioid dose during the 6 months before the incident ORAE encounter for cases. To measure opioid dose trajectories, cases were required to have at least one prescription opioid fill in the 6 months before the incident ORAE. This requirement excluded cases who had an incident ORAE within 6 months after opioid initiation and who had no prescription opioid dispensed in the 6 months before an incident ORAE. The rationale for requiring one or more opioid prescriptions is to (1) focus on cases who visit clinics to obtain prescription opioids and have a chance of being evaluated by doctors for risk of ORAE before its onset; and (2) to reduce confounding by illicit opioid use, which likely occurs among cases who had no prescription opioid use before ORAE [4]. For each case, we used an incidence density sampling approach to randomly select one control person prescribed opioids who was at risk but had not experienced an ORAE encounter by the index date of the case event. In other words, controls also had at least one dispensed opioid prescription in the 6 months before their matched date. We matched controls to cases on age, sex, and time (in days) since cohort entry because these 3 matching variables provided a sufficient number of controls for matching. We adjusted for other nonmatched confounders later in multivariable regression models.

### Prescription opioid and its dose conversion

Prescription opioids approved for use in the US between 2011 and 2018 were captured from the Medicare Part D Prescription Event files based on the National Drug Code (**S2 Table**). We excluded (1) injectable opioids because they are primarily used in inpatient settings where prescription dispensing data are not available; and (2) buprenorphine sublingual tablets and buprenorphine–naloxone combinations because they are indicated for treatment of OUD or OD.

The dose of each prescription opioid filled during 6 months before the index date was converted to an MME dose based on a standard formula—the quantity of opioids dispensed per day multiplied by the strength and the MME conversion factor [26]. We then calculated the mean daily MME dose in each month by adding the MMEs of all days with prescribed opioids dispensed during the month and then dividing by 30 days. Sensitivity analysis was conducted with the mean daily MME dose calculated at biweekly intervals.

### Statistical analysis

We used a group-based trajectory model (GBTM) to identify clusters of patients who followed a similar longitudinal pattern for prescribed opioid dose during the 6 months preceding an incident ORAE encounter for cases and matched controls. Because the monthly mean MME measure had a nonnormal distribution, to enable model convergence while retaining all MME data points, we applied natural log transformation to the MME measure and modeled log-MME as a censored normal distribution [27]. We fitted the GBTMs with 1 to 5 classes and found that a model with 4 trajectories was optimal within the recommended criteria (**S3 Table**) [27,28]. Characteristics, as well as the use of cautionary high-dose (defined as 50 daily MME) and risky high-dose (defined as 90 daily MME) in any given month, were described and compared across the 4 trajectory groups using the chi-squared test. Sensitivity analysis was performed by examining prescription opioid dose trajectories among cases with specific types of opioid encounters and their matched controls.

We used a multivariate conditional logistic regression to examine the association between the identified 4 trajectories of prescribed opioid dose and risk for ORAE in the study sample of cases and controls, adjusting for several potential confounders measured between 12 and 6 months before the index date. These confounders included demographics (race/ethnicity [defined based on Research Triangle Institute race code available in the Medicare claims database and grouped into 3 groups: White, Black, and other (including Hispanic, Asian, Pacific Islander, and Native American individuals), each with a sample size sufficient enough to ensure statistically reliable estimates], low-income subsidy status [yes/no], region [Northeast, Midwest, South, and West]), diagnosis of alcohol or tobacco use disorder, types of chronic pain conditions (musculoskeletal, neuropathic, and idiopathic pain), polypharmacy (defined as having ≥5 different medications, excluding opioids), select clinical comorbid conditions (including mental health disorders, diabetes, cardiovascular diseases, hypertension, pulmonary condition, kidney disease, gastrointestinal disorder, respiratory infections, injuries, and infections from nonsterile opioid injection, identified based on ICD codes defined in the Clinical Classifications Software (CCS) of the Healthcare Cost and Utilization Project) [29], and overall healthcare utilization (including any hospital stay, any emergency department visit, and any skilled nursing facility stay, identified based on medical claims). To account for opioid exposure time, we also calculated the duration of opioid use between opioid initiation and the day before the 6-month exposure measurement period for each individual. To account for the secular trend in national opioid prescribing, we also included the year of index date (2011 to 2018) as a linear variable in the model. **S1 Table** details diagnostic or procedure codes of the aforementioned confounders. We reported the odds ratios (ORs) and 95% confidence intervals (CIs) from the model.

We performed additional analysis by assessing trajectories of prescribed opioid doses in relation to specific types of opioid encounters (i.e., opioid misuse or dependence and opioid poisoning). We conducted a sensitivity analysis using a cohort study design with inverse probability of treatment weighting (IPTW) analysis to test the association of trajectories of prescribed opioid dose with risk of ORAEs (see details of method in **S2 Text**). **S4 Fig** showed 4 trajectories of prescribed opioid dose identified in the sensitivity analysis of a cohort design, which resemble the shapes of the 4 groups identified in the main analysis using a nested case–control design. **S5** and **S6 Tables** showed the baseline characteristics of the 4 identified trajectory groups before and after IPTW weighting, respectively, in a cohort design. After the weighting, all characteristics were balanced between the target and reference trajectory group, except for the duration of opioid use since opioid initiation, for which statistical adjustment was performed in the final weighted Cox hazard models as a sensitivity analysis. All analyses were performed using SAS 9.4, and all tests were two-sided with statistical significance set as *P* < 0.05.

## Results

**Fig 1:**
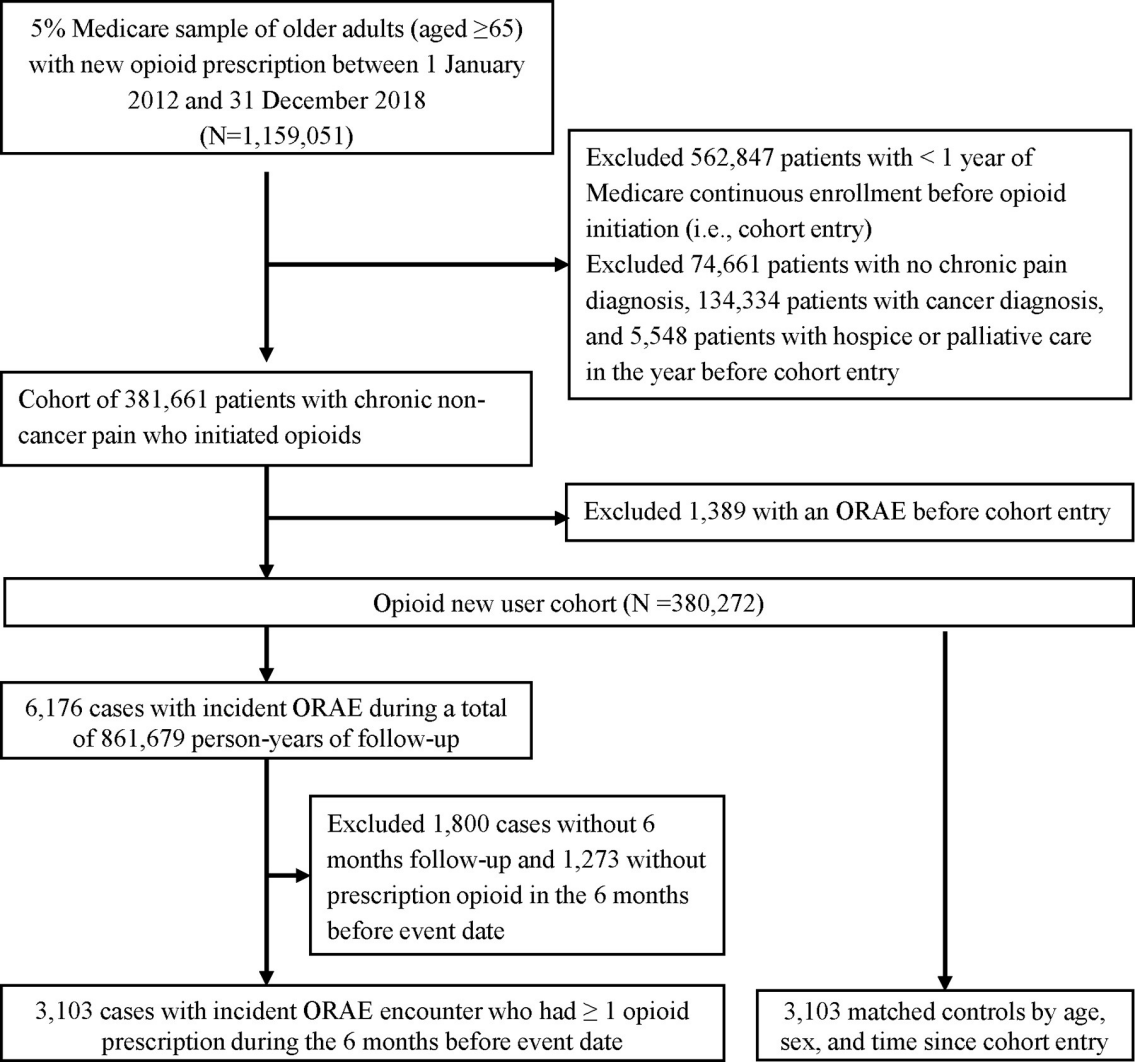
Cohort inclusion flowchart for the nested case–control study sample. ORAE, opioid-related advance event.

A cohort of 380,272 Medicare older patients with CNCP (mean [SD] age, 76.2 [7.9] years; 65.4% female; and 81.4% White) were new users of prescription opioids between 2011 and 2018 **(Table 1). Fig 1** describes the cohort inclusion and exclusion criteria. During the year before cohort entry, 9.0% had a diagnosis of tobacco or alcohol use disorder, 28.3% had mental health disorders, 44.7% had diabetes, and 59.3% had cardiovascular diseases. The majority (80.9%) had polypharmacy. Musculoskeletal pain was the most prevalent pain condition experienced in these older adults.

**Table 1:** Characteristics of the total cohort, cases who had an incident ORAE encounter, and matched controls of older adults with CNCP who were new users of prescription opioids between 2011 and 2018.

Characteristics^1^	Total cohort *N =* 380,272 (100%)	Case *n =* 3,103 (100%)	Matched controls *n* = 3,103 (100%)	*P* value
**Age** ^**2**^				NA^3^
Mean (SD)	76.2 (7.9)	77.1 (7.1)	77.1 (7.1)	
65–74	191,688 (50.4)	1,339 (43.2)	1,339 (43.2)	
75–84	122,276 (32.2)	1,225 (39.5)	1,225 (39.5)	
85+	66,308 (17.4)	539 (17.4)	539 (17.4)	
**Female** ^**2**^	248,696 (65.4)	2,192 (70.6)	2,192 (70.6)	NA^3^
**Race/ethnicity**				0.019
White	309,633 (81.4)	2,552 (82.2)	2,465 (79.4)	
Black	29,369 (7.7)	251 (8.1)	289 (9.3)	
Other^4^	41,270 (10.9)	300 (9.7)	349 (11.2)	
**Low-income subsidy status**	97,266 (25.6)	980 (31.6)	941 (30.3)	0.282
**Region**				0.003
South	153,478 (40.4)	1,341 (43.2)	1,390 (44.8)	
Northeast	66,727 (17.5)	473 (15.2)	438 (14.1)	
Midwest	90,989 (23.9)	680 (21.9)	757 (24.4)	
West	69,078 (18.2)	609 (19.6)	518 (16.7)	
**Tobacco or alcohol use disorder**	34,152 (9.0)	365 (11.8)	222 (7.2)	<0.001
**Chronic pain diagnosis**				
Musculoskeletal pain	351,602 (92.5)	2,720 (87.7)	2,506 (80.8)	<0.001
Neuropathic pain	133,023 (35.0)	1,495 (48.2)	1,053 (33.9)	<0.001
Idiopathic pain	37,231 (9.8)	825 (26.6)	409 (13.2)	<0.001
**Clinical conditions**				
Mental health disorders	107,790 (28.3)	1,094 (35.3)	822 (26.5)	<0.001
Diabetes	169,831 (44.7)	1,354 (43.6)	1,325 (42.7)	0.457
Cardiovascular diseases	225,542 (59.3)	1,834 (59.1)	1,589 (51.2)	<0.001
Hypertension	307,945 (81.0)	2,424 (78.1)	2,352 (75.8)	0.030
Pulmonary condition	243,355 (64.0)	1,811 (58.4)	1,593 (51.3)	<0.001
Kidney disease	92,115 (24.2)	797 (25.7)	662 (21.3)	<0.001
Gastrointestinal disorder	117,013 (30.8)	907 (29.2)	728 (23.5)	<0.001
Respiratory infections	105,919 (27.9)	934 (30.1)	725 (23.4)	<0.001
Injuries	92,863 (24.4)	757 (24.4)	573 (18.5)	<0.001
Infections from nonsterile opioid injection	41,864 (11.0)	298 (9.6)	250 (8.1)	0.032
**Polypharmacy**	307,515 (80.9)	2,782 (89.7)	2,668 (86.0)	<0.001
**Healthcare utilization**				
Any hospital stay	73,923 (19.4)	656 (21.1)	515 (16.6)	<0.001
Any ED visit	111,579 (29.3)	902 (29.1)	733 (23.6)	<0.001
Any SNF stay	27,585 (7.3)	238 (7.7)	164 (5.3)	<0.001

CNCP, chronic noncancer pain; ED, emergency department; NA, Not applicable; ORAE, opioid-related adverse event; SNF, skilled nursing facility.

^**1**^Characteristics of the total cohort were measured in 12 months before cohort entry (i.e., opioid initiation), and characteristics of cases and matched controls were measured between 12 and 6 months before the incident ORAE or the matched date.

^**2**^Age and sex were matched variables.

^**3**^No *P* value was reported for age and sex, variables that were already matched between cases and controls.

^**4**^Included Hispanic, Asian, Pacific Islander, and Native American individuals.

From this opioid new user cohort, we identified 6,176 patients who had an incident ORAE encounter during follow-up, yielding an incidence rate of 7.17 per 1,000 person-years. Of the 6,176 ORAE cases, 1,800 (29.1%) had the encounter within the first 6 months after prescription opioid initiation, and 1,273 (20.6%) had no prescription opioid fill in the 6 months preceding the ORAE diagnosis. This resulted in 3,103 cases with a 6-month follow-up preceding the ORAE, during which at least one opioid prescription was dispensed for dose trajectory analysis. Of 3,103 cases, 55.5% had a diagnosis of opioid misuse or dependence, 45.1% had a diagnosis of opioid poisoning, and only 0.06% had a diagnosis of adverse effects of opioids. **Table 1** gives the characteristics of the 3,103 cases and 3,103 matched control patients.

**Fig 2:**
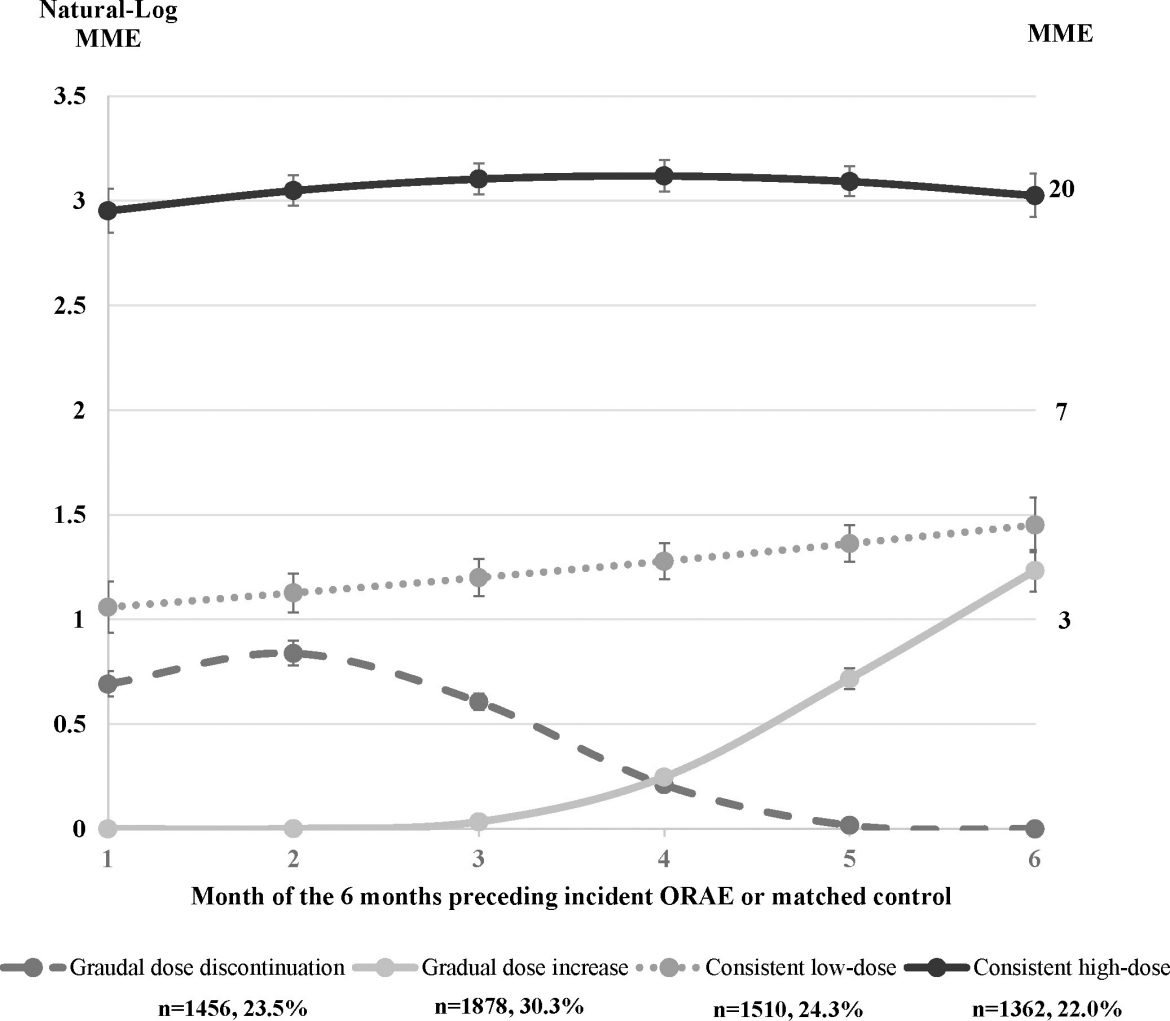
Trajectories of mean daily MME dose prescribed in each month for the 6 months preceding an incident encounter of ORAE for cases or matched controls of older adults. Lines represent types of dose trajectory group, and for each line, each point represents the mean daily MME of prescription opioids per month. The scale on the left and right side of the figure is the natural logarithm of MME and actual MME, respectively. The error bar represents the standard deviation of the natural logarithm transformed MME. MME, morphine milligram equivalent; ORAE, opioid-related adverse event.

We identified distinct opioid dose trajectories before an incident ORAE encounter or matched date in controls (**Fig 2**). Four trajectories, categorized based on their mean daily MME use of prescription opioids per month, included patients with gradual dose discontinuation (from ≤3 to 0 daily MME, consisting of 23.5% of the study sample), gradual dose increase (from 0 to >3 daily MME, 30.3%), consistent low-dose use (between 3 and 5 daily MME, 24.3%), and consistent moderate-dose use (>20 daily MME, 22.0%). The dose trajectory groups differed significantly for most demographics as well as select pain and clinical conditions (**Table 2).**

**Table 2:** Characteristics of defined trajectories of prescribed opioid dose within 6 months before an incident diagnosis of ORAE or matched controls.

Characteristic	Percentage (%) of cases and matched controls with ≥1 prescription opioid filled within 6 months before ORAEs (*n =* 6,206)					
	Gradual dose discontinuation	Gradual dose increase	Consistent low-dose	Consistent moderate-dose		*P* value for group difference
Total sample size	1,456 (100%)	1,878 (100%)	1,510 (100%)	1,362 (100%)		
**Age, y**						.001
65–74	695 (47.7)	786 (41.9)	614 (40.7)	583 (42.8)		
75–84	557 (38.3)	755 (40.2)	612 (40.5)	526 (38.6)		
85+	204 (14.0)	337 (17.9)	284 (18.8)	253 (18.6)		
**Female**	1,002 (68.8)	1,326 (70.6)	1,095 (72.5)	961 (70.6)		.179
**Race/ethnicity**						.015
White	1,171 (80.4)	1,513 (80.6)	1,186 (78.5)	1,147 (84.2)		
Black	128 (8.8)	162 (8.6)	149 (9.9)	101 (7.4)		
Other^1^	157 (10.8)	203 (10.8)	175 (11.6)	114 (8.4)		
**LIS status**	376 (25.8)	473 (25.2)	512 (33.9)	560 (41.1)		< .001
**Region**						< .001
South	638 (43.8)	801 (42.7)	686 (45.4)	606 (44.5)		
Northeast	188 (12.9)	312 (16.6)	221 (14.6)	190 (14.0)		
Midwest	361 (24.8)	446 (23.7)	336 (22.3)	294 (21.6)		
West	269 (18.5)	319 (17.0)	267 (17.7)	272 (20.0)		
**Tobacco or alcohol use disorder**	115 (7.9)	150 (8.0)	162 (10.7)	160 (11.7)		< .001
**Chronic pain diagnosis**						
Musculoskeletal pain	1,196 (82.1)	1,454 (77.4)	1,325 (87.7)	1,251 (91.9)		< .001
Neuropathic pain	522 (35.9)	658 (35.0)	674 (44.6)	694 (51.0)		< .001
Idiopathic pain	186 (12.8)	226 (12.0)	301 (19.9)	521 (38.3)		< .001
**Clinical conditions**						
Mental health disorders	371 (25.5)	498 (26.5)	491 (32.5)	556 (40.8)		< .001
Diabetes	640 (44.0)	761 (40.5)	686 (45.4)	592 (43.5)		.030
CVD	726 (49.9)	1,009 (53.7)	883 (58.5)	805 (59.1)		< .001
Hypertension	1,107 (76.0)	1,405 (74.8)	1,189 (78.7)	1,075 (78.9)		.010
Pulmonary condition	773 (53.1)	991 (52.8)	887 (58.7)	753 (55.3)		.002
Kidney disease	354 (24.3)	400 (21.3)	388 (25.7)	317 (23.3)		.020
Gastrointestinal disorder	359 (24.7)	461 (24.5)	421 (27.9)	394 (28.9)		.008
Respiratory infections	355 (24.4)	459 (24.4)	440 (29.1)	405 (29.7)		< .001
Injuries	300 (20.6)	372 (19.8)	330 (21.9)	328 (24.1)		.030
Infections due to nonsterile opioid injection	109 (7.5)	147 (7.8)	134 (8.9)	158 (11.6)		< .001
**Polypharmacy**	1,250 (85.9)	1,554 (82.7)	1,376 (91.1)	1,270 (93.2)		< .001
**Healthcare utilization**						
Any hospital stay	266 (18.3)	312 (16.6)	318 (21.1)	275 (20.2)		.005
Any ED visit	371 (25.5)	491 (26.1)	407 (27.0)	366 (26.9)		.781
Any SNF stay	88 (6.0)	80 (4.3)	118 (7.8)	116 (8.5)		< .001
**Types of Prescription opioid**						< .001
Short-acting only	1,441 (99.0)	1,822 (97.0)	1,422 (94.2)	1,047 (76.9)		
Long-acting only	--	44 (2.3)	79 (5.2)	250 (18.4)		
Both	--	--	--	65 (4.8)		
**High dose**						< .001
≥50 daily MME in a month	16 (1.1)	22 (1.2)	59 (3.9)	385 (28.3)		
≥90 daily MME in a month	--	--	--	9.8		
**Duration of opioid use since opioid initiation, days** Mean (SD)	69.3 (123.1)	50.4 (97.2)	168.4 (226.1)	420.4 (377.6)	< .001	

CVD, cardiovascular disease; ED, emergency department; LIS, low-income subsidy; MME, morphine milligram equivalent; ORAE, opioid-related adverse event; SNF, skilled nursing facility.

^**1**^Included Hispanic, Asian, Pacific Islander, and Native American individuals.

Compared with controls, cases had a lower proportion of patients with gradual dose discontinuation (11.8% versus 35.2%, *P* < 0.001) but a higher proportion with consistent moderate dose (31.6% versus 12.3%, *P* < 0.001) (**Table 3**). Overall, only 2.4% of older patients were prescribed a mean daily dose of 90 mg MME or more during any month of the 6 months before the index date. Sensitivity analysis with mean daily MME use of prescription opioids calculated at the biweekly interval showed similar dose trajectories (**S1 Fig**). We found similar prescription opioid dose trajectories among cases with opioid misuse or dependence encounters and their controls (**S2 Fig**) and among cases with opioid poisoning encounters and their controls (**S3 Fig**).

**Table 3:** Distribution of prescription dose trajectories group and monthly dose in MME in the study sample, cases, and matched controls.

	Study Sample *N* = 6,206 (100%)	Cases *N =* 3,103 (100%)	Controls *N =* 3,103 (100%)	*P* value
**Dose trajectory group**				*P* < 0.001
Gradual dose discontinuation group	1,456 (23.5)	365 (11.8)	1,091 (35.2)	
Gradual dose increase group	1,878 (30.3)	931 (30.0)	947 (30.5)	
Consistent low-dose group	1,510 (24.3)	826 (26.6)	684 (22.0)	
Consistent moderate-dose group	1,362 (22.0)	981 (31.6)	381 (12.3)	
**Monthly MME (%)**				
At least 1 month with ≥50 daily MME	(482) 7.8	(410) 13.2	72 (2.3)	*P* < 0.001
At least 1 month with ≥90 daily MME	(150) 2.4	(132) 4.3	18 (0.6)	*P* < 0.001

MME, morphine milligram equivalent.

In adjusted multivariable conditional logistic regression analysis, prescription opioid dose trajectories were independently associated with risk for an ORAE encounter (**Table 4**). Compared to patients with gradual dose discontinuation, those with graduate dose increase had a 3.4-fold (95% CI 2.8 to 4.0; *P* < 0.001), those with consistent low dose had a 3.8-fold (95% CI 3.2 to 4.6; *P* < 0.001), and those with consistent moderate dose had an 8.5-fold (95% CI 6.8 to 10.7; *P* < 0.001) increased risk for having an incident ORAE encounter, after adjustment for covariates. Stratification analysis by specific types of opioid encounters showed similar results, with the gradual dose increase, consistent low dose, and consistent moderate dose group having an increased risk for opioid misuse/dependence or poisoning when compared to the group with decreasing dose trajectory (**S4 Table**).

**Table 4:** Unadjusted and adjusted association between trajectories of prescription opioid dose and risk for ORAEs.

Variables	Cases vs. matched controls			
	Unadjusted OR (95% CI)	*P* value	Adjusted^1^ OR (95% CI)	*P* value
**Opioid dose trajectory group**				
Gradual dose discontinuation group	1.00 Reference		1.00 Reference	
Gradual dose increase group	3.09 (2.63–3.64)	<0.001	3.36 (2.83–4.00)	<0.001
Consistent low-dose group	3.82 (3.22–4.54)	<0.001	3.81 (3.17–4.58)	<0.001
Consistent moderate-dose group	8.28 (6.86–9.99)	<0.001	8.53 (6.79–10.70)	<0.001
**Race/ethnicity**				
White	1.00 Reference		1.00 Reference	
Black	0.84 (0.70–1.00)	0.050	0.88 (0.70–1.10)	0.273
Other^2^	0.83 (0.71–0.98)	0.026	0.97 (0.79–1.19)	0.765
**Low-income subsidy status** (yes vs. no as the reference)	1.06 (0.95–1.18)	0.287	0.89 (0.77–1.03)	0.117
**Region**				
South	1.00 Reference		1.00 Reference	
Northeast	1.12 (0.96–1.30)	0.144	1.15 (0.96–1.38)	0.121
Midwest	0.93 (0.82–1.06)	0.300	0.97 (0.83–1.13)	0.670
West	1.21 (1.06–1.39)	0.006	1.24 (1.05–1.47)	0.002
**Tobacco or alcohol use disorder** (yes vs. no)	1.74 (1.46–2.08)	<0.001	1.48 (1.30–1.68)	0.030
**Chronic pain diagnosis**				
Musculoskeletal pain (yes vs. no)	1.69 (1.47–1.94)	<0.001	1.21 (1.02–1.43)	0.035
Neuropathic pain (yes vs. no)	1.84 (1.65–2.05)	<0.001	1.48 (1.30–1.68)	<0.001
Idiopathic pain (yes vs. no)	2.44 (2.13–2.80)	<0.001	1.58 (1.34–1.87)	<0.001
**Clinical conditions**				
Mental health conditions (yes vs. no)	1.51 (1.35–1.69)	<0.001	1.19 (1.03–1.36)	0.014
Diabetes (yes vs. no)	1.04 (0.94–1.15)	0.451	0.88 (0.77–1.01)	0.067
Cardiovascular disease (yes vs. no)	1.39 (1.26–1.54)	<0.001	1.16 (1.02–1.33)	0.028
Hypertension (yes vs. no)	1.15 (1.02–1.30)	0.026	1.00 (0.85–1.17)	0.973
Pulmonary condition (yes vs. no)	1.33 (1.20–1.47)	<0.001	1.08 (0.93–1.26)	0.294
Kidney disease (yes vs. no)	1.28 (1.14–1.45)	<0.001	1.31 (1.12–1.52)	<0.001
Gastrointestinal disorder (yes vs. no)	1.35 (1.20–1.51)	<0.001	1.10 (0.95–1.28)	0.185
Respiratory infections (yes vs. no)	1.40 (1.25–1.56)	<0.001	1.17 (1.00–1.37)	0.056
Injuries (yes vs. no)	1.42 (1.26–1.61)	<0.001	1.03 (0.87–1.22)	0.741
Infections from nonsterile opioid injection (yes vs. no)	1.21 (1.02–1.45)	0.032	0.91 (0.73–1.12)	0.360
**Medication utilization**				
Polypharmacy (yes vs. no)	1.34 (1.22–1.67)	<0.001	0.96 (0.78–1.17)	0.660
**Healthcare utilization**				
Any hospital stay (yes vs. no)	1.35 (1.19–1.54)	<0.001	0.94 (0.77–1.13)	0.488
Any ED visit (yes vs. no)	1.33 (1.19–1.54)	<0.001	1.03 (0.88–1.20)	0.672
Any SNF stay (yes vs. no)	1.49 (1.21–1.83)	<0.001	1.00 (0.76–1.31)	0.995
Duration of opioid use since opioid initiation (per 30 days)	1.04 (1.03–1.04)	<0.001	0.99 (0.98–1.00)	0.061

CI, confidence interval; ED, emergency department; OR, odds ratio; ORAE, opioid-related adverse event; SNF, skilled nursing facility.

^**1**^Adjusted for race/ethnicity, low-income subsidy status, region, tobacco or alcohol use disorder, chronic pain diagnoses, clinical conditions, medication utilization, healthcare utilization, and the year of the index date.

^**2**^Included Hispanic, Asian, Pacific Islander, and Native American individuals.

Our main findings remained robust in the sensitivity analysis using a cohort study design. The increased risk of ORAE among patients with the gradual dose increase (adjusted hazard ratio [HR] = 4.4; 95% CI 3.8 to 5.1; *P* < 0.001), consistent low dose (aHR = 1.9; 95% CI 1.6 to 2.1; *P* < 0.001), and consistent moderate dose (aHR = 5.7; 95% CI 5.0 to 6.5; *P* < 0.001), as compared to those with the gradual dose discontinuation, persisted in a cohort design (**S7 Table**). Further stratification by the duration of follow-up revealed a higher risk of ORAE in the earlier months (i.e., first or second month) of the follow-up defined in the cohort design as a sensitivity analysis (**S7 Table**).

## Discussion

In this sample of older Medicare beneficiaries, we found that 4 trajectories of opioid dose prescribed during 6 months before the incident ORAE diagnosis or matched date emerged: gradual dose discontinuation (from ≤3 to 0 daily MME), gradual dose increase (from 0 to >3 daily MME), consistent low dose (between 3 and 5 daily MME), and consistent moderate dose (>20 daily MME). Overall, few older patients (<5%) were prescribed a mean daily dose of ≥90 daily MME before diagnosis or matched date. Compared to older patients with gradual dose discontinuation, those with gradual dose increase, consistent low dose, and consistent moderate dose use group had a higher risk of ORAE. The findings were consistent in a sensitivity analysis using a cohort design.

Across the 4 identified groups, we observed a low dose range (mean daily dose between 0 to 20 daily MME) of prescribed opioids and less than 5% of older patients, the majority of whom were among the cases, receiving doses at or above the dose threshold of 90 daily MME. Compared to evidence observed in younger adults with ORAE [25], a lower dose range (0 to 20 daily MME versus 3 to 150 daily MME) and a lower proportion with ≥90 daily MME (2.4% versus 28.8%) was observed among older adults with a similar diagnosis of ORAE, suggesting that there is a unique opioid dosage pattern preceding the incident ORAE encounter in the older population.

The mechanisms by which low to moderate doses of prescribed opioids were associated with increased risk of ORAEs among older patients may be complex and can possibly be explained by 2 major pathways. First, older patients may be more susceptible to opioid side effects at lower doses. While no empirical data or clinical consensus exists on a dose threshold above which opioids are considered harmful for older patients, evidence from the general population has suggested that increased risk of OD may occur at a dose as low as 20 mg/day MME [30]. The other pathway that may explain our observed associations is the use of illicit opioids, which cannot be captured with our data sources, to supplement the low-to-moderate dose of prescribed opioids. Recent reports suggest an emerging transition from prescription opioids to illicit opioids owing to increasingly restricted access to prescription opioids [31–33]. It is possible that the gradual dose discontinuation group identified in this study might have sought illicit opioids to achieve pain control or to enhance euphoric effects, putting them at higher risk for ORAEs, compared to other groups of prescription opioid dose. Yet, this assumption was not supported by our data where a lower risk of ORAEs was observed in the group with gradual dose discontinuation versus other identified prescription opioid dose groups. Further studies that examine whether illicit opioid use varies across the 4 identified prescribed opioid dose trajectories are needed to assist interpretations of our study findings.

Of note is our finding that over 1 in 4 (28.3%) older patients gradually discontinued their prescription opioids in 6 months before an ORAE or matched date for controls. The opioid discontinuation was much higher in the matched controls (35.2%) than in cases (11.8%). Reasons for more controls undergoing opioid discontinuation are unclear but could be because of improved pain control, opioid ineffectiveness, or adverse opioid side effects [34]. Opioid discontinuation or dose reduction, particularly among long-term users, is among the recommendations from the 2016 CDC opioid guidance [14]. While the CDC guideline did not support abrupt tapering and sudden discontinuation of prescription opioid dose, these clinical practices were reported for patients with chronic pain and have been implicated as a contributor to unintended consequences, including ORAEs [2], OD and deaths [35], and suicidal ideation or attempts [35,36]. The US Department of Health and Human Services in 2019 issued a new clinical guide on how to appropriately reduce dose or discontinue long-term opioid analgesics, emphasizing the importance of assessing the risks and benefits of such practices [37]. In the present study, we focused on opioid-naïve older adults and found that those with gradual dose discontinuation (versus those with gradual dose increase or those with low-to-moderate dose use) had decreased risk for ORAE. It is worth noting that the gradual dose discontinuation group identified in the present study was named based on the trajectory shape, and the definition of our gradual dose discontinuation is not the same as that defined by the CDC, which involves opioid dose reduction of 10% per week after opioid use for weeks to months and 10% per month following opioid use for >1 year [37]. Whether our association findings can be seen for older adults with long-term opioid therapy requires further investigations to understand the benefits and harms of discontinuation of long-term opioid analgesics.

The present study has several noteworthy strengths. The use of a nationally representative sample of older adults who are Medicare beneficiaries from 2011 to 2018 provides population-based data that reflect current opioid prescribing practices and supplements current literature on prescription opioid dose patterns relevant to older adults at risk for ORAEs. The national data also provide a sufficient number of older adults with an incident ORAE, allowing adequate power to detect the association between trajectories of prescription opioid dose and risk for ORAE.

There are also several limitations to note. First, this study allows for establishing an association but not causation between prescription opioid dose trajectories and risk for ORAEs. Second, illicit opioid use, a growing concern in the opioid epidemic, was not captured in our data, limiting our ability to clarify the safe dose threshold of prescribed opioids for older adults. Third, our analysis of prescription dispensing data confirms receipt of medications and not medication use. Fourth, Medicare administrative claims data lack information on pain severity, which is the key factor associated with selection into opioid treatment. Fifth, while several opioid endpoints defined in claims data have been validated against medical chart review [38–40], the validity of ORAE is unclear and warrants further research. Sixth, the present study did not measure the risk of adverse events associated with opioid tapering such as increased pain, insomnia, mental and physical function, and suicide. Seventh, our findings can only be generalized to Medicare fee-for-service beneficiaries with CNCP. Finally, our study excluded patients who had an incident diagnosis of ORAEs but had no prescription opioid fill during the 6 months before the diagnosis. This group may present different opioid risk profiles, and understanding risk factors beyond prescription opioid use is important to identify this high-risk subgroup of older patients.

Our findings have important clinical implications. The CDC-recommended 90 mg/day MME as the high-risk opioid dose threshold may be impractical to detect older adults at risk for ORAE. Only 5% of cases received prescribed opioid doses at or above 90 daily MME before diagnosis, leaving most cases undetected. Since 2013, the CMS required its Medicare Part D sponsors to closely monitor high-risk beneficiaries whose prescribed opioid dose was at or above 120 daily MME, and in recent years, they aligned the risky dose threshold to be consistent with the CDC-recommended 90 daily MME [41]. Prior studies have questioned the utility of using 90 daily MME in detecting patients at risk of ORAEs [21]. Echoing this finding, our study suggests that additional clinical markers to predict illicit opioid use are needed to identify older adult patients at high risk for ORAEs, particularly during the new era of increasingly restricted access to prescription opioids.

## Conclusions

In this sample of older patients who are Medicare beneficiaries, 4 prescription opioid dose trajectories were identified, with most prescribed doses below 90 daily MME within 6 months before an incident ORAE encounter or matched date. An increased risk for ORAE was observed among older patients who had a gradual increase in dose or among those who had a consistent low-to-moderate dose of prescribed opioids when compared to patients with opioid dose discontinuation. Our main findings remained robust in the sensitivity analysis using a cohort study design. Further studies are needed that examine whether older patients are susceptible to low opioid doses.

## Supporting information

S1 STROBE Checklist Strengthening the reporting of observational studies in epidemiology (STROBE) checklist. (DOC)

S1 Text Prespecified analysis plan. (DOCX)

S2 Text Sensitivity analysis using a cohort study design. (DOCX)

S1 Table ICD-9-CM or ICD-10-CM Codes and procedures for disease conditions and service care considered in the study. (DOCX)

S2 Table Study prescription opioids approved by the US Food and Drug Administration for use in the US market from 2011 to 2018. (DOCX)

S3 Table Criteria used to decide an optimal solution for the number of latent groups among case and control patients. (DOCX)

S4 Table Adjusted association of trajectories of prescription opioid dose with risk for specific types of ORAEs. ORAE, opioid-related adverse event. (DOCX)

S5 Table Sensitivity analysis of characteristics of eligible older patients, overall and by defined trajectories of prescribed opioid dose, in a cohort design. (DOCX)

S6 Table Sensitivity analysis of characteristics of eligible older patients with defined trajectories of prescribed opioid dose after inverse propensity of treatment weighting in a cohort design. (DOCX)

S7 Table Sensitivity analysis of cohort study for the association of defined prescribed opioid dose trajectories with risk for ORAEs during 180-day follow-up. ORAE, opioid-related adverse event.
(DOCX)

S1 Fig Trajectories of mean daily MME dose prescribed in biweekly within 6 months preceding the incident diagnosis of ORAEs for cases and matched controls of older patients. MME, morphine milligram equivalent; ORAE, opioid-related adverse event.
(TIFF)

S2 Fig Trajectories of mean daily MME dose prescribed in each month within 6 months preceding an incident diagnosis of opioid misuse or dependence and matched controls of older patients. MME, morphine milligram equivalent; ORAE, opioid-related adverse event.
(TIFF)

S3 Fig Trajectories of mean daily MME dose prescribed in each month within 6 months preceding an incident diagnosis of opioid poisoning and matched controls of older patients. MME, morphine milligram equivalent; ORAE, opioid-related adverse event.
(TIFF)

S4 Fig Sensitivity analysis of trajectories of mean daily MME dose prescribed in each month over a randomly selected 6 months period in a cohort of older patients. MME, morphine milligram equivalent.
(TIFF)

## Abbreviations

AHRQ: Agency for Healthcare Research and Quality
aOR: adjusted odds ratio
CDC: Centers for Disease Control and Prevention
CI: confidence interval
CMS: Centers for Medicare and Medicaid Services
CNCP: chronic noncancer pain
GBTM: group-based trajectory model
HR: hazard ratio
IPTW: inverse probability of treatment weighting
MME: morphine milligram equivalent
OR: odds ratio
ORAE: opioid-related adverse event
OUD: opioid use disorder
