# Supplementary material for: Trajectories of prescription opioid dose and risk of opioid-related adverse events among older Medicare beneficiaries in the United States: A nested case–control study
Source: PLoS Med. 2022 Mar 15;19(3):e1003947. doi: 10.1371/journal.pmed.1003947 (PMC8923459; doi:10.1371/journal.pmed.1003947)
Supplement: S1 Text — (DOCX) [file pmed.1003947.s002.docx]

**Background:**

Despite an abundance of efforts to combat the national opioid epidemic, medical utilization due to opioid dependence, abuse, or poisoning among older adults has grown disproportionally. To accurately identify the high-risk elderly groups and implement effective preventive measures, it is important to understand the etiology of opioid-related hospitalizations, with a special emphasis on elderly-specific risk factors. This study will assess elderly high-risk prescription opioid use patterns, predisposing and prognostic factors, and their associations with opioid-related hospitalizations. To understand the progress from prescription opioid initiation to the development of opioid-related hospitalization outcomes, the target population is Medicare beneficiaries who initiate prescription opioids at an older age (aged 65+) included in a nationally representative sample of Medicare beneficiaries from 2011-2018.

Many studies have explored prescription opioid use patterns and their association with adverse opioid events, including opioid misuse, related overdoses, and deaths among non-elderly^1-4^ or mixed populations[.](#_bookmark6)^5-7^ Only a few existing studies focused on elderly populations from a limited or non-representative sample.^8-10^ It remains unclear whether the employed metrics of high-risk prescription opioid use (e.g., high daily dose) from non-elderly populations are applicable to older adults who have a different threshold for adverse opioid outcomes due to declined renal/hepatic function, other comorbidities, and poly-pharmacy.^9,11^ In addition, none of the prior studies was conducted during the new era of increasingly restricted access to prescription opioids. Given the increasing transparency of prescription opioid access in Prescription Drug Monitoring Programs and other initiatives to reduce access to prescription opioids, early refills or multiple provider criteria may have decreasing utility in identifying high-risk users.^12,13^ Further, the only existing elderly studies evaluated opioid use cross-sectionally, limiting assessments of the progression of opioid use to adverse opioid outcomes. ^8-10^ Understanding prescription opioid use trajectories is important for identification and intervention for high-risk groups*.* We propose a longitudinal assessment of opioid utilization to identify trajectories from opioid initiation to high-risk use and manifestation of opioid-related hospitalizations in older adults.

**Overall impact**: The contribution of the research is significant because the results will provide an understanding of prescription opioid use and risk factors of opioid-related hospitalizations in older adults. The findings will support the development of an opioid risk tool for identification of older adults at risk for adverse opioid outcomes.

**Study Aim:** Examine trajectories of prescription opioid use and their association with opioid-related adverse events (ORAE) after initiation among opioid-naïve Medicare older adults.

**Research design:** Nested case-control to emulate the real-world clinical practice where a limited time window of patient history data is often available for routine clinical assessment

**Data source:** 5% random national sample of Medicare beneficiaries from 2011- 2018 will constitute the study population. We chose Medicare data because 1) they represent older adults ≥ 65 years^14^[;](#_bookmark31) 2) capture in- and outpatient encounters associated with opioid-related hospitalizations; and 3) provide a comprehensive account of prescription opioid and other prescription use reimbursed under Medicare Part D.15 Medicare Parts A & B clinical encounter data include detail on procedures and diagnoses (coded using *the International Classification of Disease, 9th or 10th Revision, Clinical Modification, ICD-9-CM or ICD-10-CM).* Part D data include information on dispensed drug names, days’ supply, dosage form, and fill dates. Beneficiary enrollment and demographics are also available.

**Sample selection:**

*Step 1: create an opioid-naïve cohort by applying the following criteria:*

1. Initiated prescription opioids during 1/1/2012-12/31/2017
   1. Opioid initiation is defined as no opioid prescription dispensed within 12 months preceding the date of the first opioid prescription fill (i.e., “opioid initiation date”).
2. Aged ≥65 years on the opioid initiation date
3. Had at least one of these three chronic pain conditions: a) musculoskeletal pain; b) neuropathic pain; or c) idiopathic pain diagnosed within 12 months before the opioid initiation date
4. Had survived throughout 12 months before the opioid initiation date
5. Had continuously enrolled in Parts A/B/PDP and no HMO/EMP coverage throughout the 12 months before the opioid initiation date
6. Had no cancer, hospice care, and palliative care during the 12 months before the opioid initiation date
7. Had no opioid-related adverse events (ORAE, i.e., outcome of interest) during the 12 months before the opioid initiation date

*Follow-up*: From opioid initiation date until an ORAE event, cancer diagnosis, receiving hospice care, death, Medicare disenrollment, or study end (12/31/2018)

*Step 2: select cases and controls from the cohort created in step 1*

1. Select ***incident* cases** who
   1. had An ORAE during the follow-up, and
   2. had at least 6 months of follow-up *before* the date of incident ORAE outcome, and
   3. had at least one prescription opioid during the 6-month pre-ORAE period to measure opioid dose trajectories preceding the ORAE outcome
2. For each case, select ***1 control*** using incidence-density sampling and match by age, gender, and time since opioid initiation (i.e., cohort entry).
   1. Assign the date of matched controls on the event date for the case

Present sample selection in a flow chart (i.e., Figure 1)

**Prescription opioids** (*key exposure*): Study prescription opioids include those approved by the US food and drug administration for use in the US market between 2005 and 2018. We capture the studied prescription opioids through the Medicare Part D prescription event files. We excluded injectable opioids primarily used in inpatient settings where dispensing information is unavailable due to capitation-based reimbursement, rectal dosage forms, which are rarely used, and buprenorphine because it is mostly used for the treatment of opioid use disorder or overdose. We will convert the dose of each prescribed opioid fill to morphine milligram equivalent using a standard formula used by the Center for Medicare and Medicaid Services.^16^

**Covariates:** We measured covariates at the baseline, defined as a 6-month period between -12 to -7 months before the event date for cases and matched date for controls. The covariates of interest include:

1. *Demographics*: age (65-74, 75-84, 85+), sex, race/ethnicity (Whites, Blacks, and Others), low-income subsidy status (Yes vs no), place of residence (based on five-digit ZIP codes and classified as South, Northeast, Midwest, and West), all of which are derived from Medicare Beneficiary Summary File.
2. *Health status*: Tobacco or alcohol use disorder (Yes vs no, assessed based on the diagnostic algorithm for both conditions developed by the Chronic Condition Warehouse, <https://www2.ccwdata.org/web/guest/condition-categories>)
3. *Chronic pain diagnosis*: classified as musculoskeletal pain (Yes vs no), neuropathic pain (Yes vs no), or idiopathic pain (Yes vs no). See ICD-9 or ICD-10 codes in the supplemental file. Note that an individual can have more than one type of chronic pain conditions.
4. *Clinical conditions* that may affect opioid treatment, including mental health disorders, diabetes, cardiovascular diseases, hypertension, pulmonary condition, kidney disease, gastrointestinal disorder, respiratory infections, injuries, and infections from non-sterile opioid injection.
5. *Health care utilization*: polypharmacy (defined as use of > 4 distinct generic drugs simultaneously), any hospital stay (yes vs no), any emergency department visit (yes vs no), any skilled nursing facility stay (yes vs no)

**Data analyses:** Our overall analytic approach include descriptive analyses, group-based trajectory modeling, and multivariable conditional logistic models. All tests are at two-sided with statistical significance at P < .05. Our analytical plan is detailed as follows:

1. Descriptive analysis:

Table 1: Describe baseline characteristics of the cohort of opioid-naïve Medicare older adults, overall and selected cases and controls

Table 2: Describe baseline characteristics of defined trajectories of prescription opioid use metrics among the sample of selected cases and controls

1. Group-based trajectory modeling (GBTM), which is detailed in the following section.

Figure 2: Trajectories of mean daily morphine equivalent milligrams (MME) dose prescribed in monthly intervals for the 6 months before the incident ORAE come for cases and matched date for controls

Sensitivity analyses:

Supplement figure: Trajectories of Mean Daily Morphine Milligram Equivalent (MME) Dose Prescribed in *Bi-weekly Interval* Within 6 Months Preceding Incident Diagnosis of Opioid-Related Adverse Events (ORAEs) for Cases and Matched Controls of Older Patients.

Supplement figure: Trajectories of Mean Daily Morphine Milligram Equivalent (MME) Dose Prescribed in each Month Within 6 Months Preceding an *Incident Diagnosis of Opioid Abuse or Dependence and Matched Controls of Older Patients***.**

**Supplement figure:** Trajectories of Mean Daily Morphine Milligram Equivalent (MME) Dose Prescribed in each month Within 6 Months Preceding an *Incident Diagnosis of Opioid Poisoning and Matched Controls of Older Patients***.**

1. Multivariable conditional logistic models:

Table 3: Unadjusted and adjusted association between identified trajectories of prescription opioid use metrics and risk for opioid-related adverse events (ORAEs)

Sensitivity analysis:

Supplement table: Unadjusted and adjusted Association of Trajectories of Prescription Opioid Dose with Risk for Specific Types of Opioid-Related Adverse Events (ORAEs)

**Group-based trajectory modeling (GBTM):**

In the sample of opioid-naïve older adults with ORAE event and matched controls, we used GBTM, a latent class analysis, to describe the natural history of prescription opioid dose within 6 months before the event onset and matched date.^17^ The change of prescription opioid dose over time is its developmental trajectory. According to the assumption of GBTM, the study sample (including both cases and matched controls) has a finite number of clusters of individuals, and each cluster follows approximately a similar trajectory of opioid dose before the event diagnosis. GBTM provides an empirical means of identifying clusters of individuals following typical and atypical development, which can help clinicians conceptualize the change of clinical conditions or treatments.^17^

**GBTM estimation**s are generated by maximum likelihood estimation. Equation 1 describes the likelihood of an individual’s observed repeated prescription opioid dose is composed of two elements $\pi_{j}$, which denotes the probability of trajectory group membership*; and* $P^{j}\left( Y_{i} \right)$, which denotes the probability of the observed outcome data given group membership

1. $P\left( Y_{i} \right)$=$\sum_{j} \pi_{j}P^{j}(Y_{i})$ ………………………………. Equation 1^17^

$Y_{i}$= prescription opioid dose trajectory data for an individual *i* over the 6 months before the incident ORAE diagnosis for cases and matched date for controls

$P^{j}\left( Y_{i} \right)$= probability of $Y_{i}$ if belonging to group *j*

$\pi_{j}$=probability of trajectory group membership *j=1,…, J*

The group membership probabilities are estimated by a multinomial logit function. The conditional probability of $Y_{i}$ given group membership j is indexed by the unknown parameter vector j, which also determines the shape of the group-specific trajectory. The conditional probability of $Y_{i}$ (i.e., outcome measure) is determined with a polynomial function of time. Depending on the distribution of the outcome measure, GBTM can model the data in various forms, including normal, censored normal, binary, or Poisson distribution. Because our prescription opioid dose data clusters vary within and across individuals over time, the data of prescription opioid dose was not normally distrusted. Following prior studies, we addressed the non-normally distributed continuous data by applying the natural log transformation of our data. We then model the log-transformed opioid dose trajectory data using censored normal distribution, one of the function forms available in GBTM.

**Evidence of clusters in GBTM**: We use GBTM to map the longitudinal pattern of prescription opioid use and to identify clusters of initiators with similar trajectories using PROC TRAJ in SAS version 9.4. (download from [www.andrew.cmu.edu/~bjones](http://www.andrew.cmu.edu/~bjones)) ^17^ Following recommended procedures, we will test the model with three different polynomial forms, including linear, quadratic, and cubic terms to determine the best trajectory shape that fits the prescription opioid dose data. We will then determine the optimal number of trajectory groups based on (1) Bayesian information criteria (BIC) and Akaike information criterion (AIC), with a lower BIC/AIC indicating a better model fit; (2) model adequacy, evidenced by an average posterior probability of at least 0.7 in each group identified; (3) sufficient group size constituting at least 5% of the total sample; and (4) clinical relevance. After identifying trajectory groups for each opioid use metric and the composite measure, we will use chi-square tests to explore whether baseline patient characteristics and pain diagnoses differ among groups.

**Reference**

1. Sullivan MD, Edlund MJ, Fan MY, Devries A, Brennan Braden J, Martin BC. Risks for possible and probable opioid misuse among recipients of chronic opioid therapy in commercial and medicaid insurance plans: The TROUP Study. Pain. 2010;150(2):332-339.

2. Cochran BN, Flentje A, Heck NC, Bos JV, Perlman D, Torres J, et al. Factors predicting development of opioid use disorders among individuals who receive an initial opioid prescription: mathematical modeling using a database of commercially-insured individuals. Drug Alcohol Depend*.* 2014;138:202-208.

3. Gwira Baumblatt JA, Wiedeman C, Dunn JR, Schaffner W, Paulozzi LJ, Jones TF. High-risk use by patients prescribed opioids for pain and its role in overdose deaths. JAMA Intern Med. 2014;174(5):796-801.

4. Edlund MJ, Martin BC, Fan MY, Devries A, Braden JB, Sullivan MD. Risks for opioid abuse and dependence among recipients of chronic opioid therapy: results from the TROUP study. Drug Alcohol Depend*.* 2010;112(1-2):90-98.

5. Dufour R, Joshi AV, Pasquale MK, Schaaf D, Mardekian J, Andrews GA, et al. The prevalence of diagnosed opioid abuse in commercial and Medicare managed care populations. Pain Pract. 2014;14(3): E106-115.

6. Edlund MJ, Steffick D, Hudson T, Harris KM, Sullivan M. Risk factors for clinically recognized opioid abuse and dependence among veterans using opioids for chronic non-cancer pain. Pain. 2007;129(3):355-362.

7. Carey CM, Jena AB, Barnett ML. Patterns of Potential Opioid Misuse and Subsequent Adverse Outcomes in Medicare, 2008 to 2012. Ann Intern Med. 2018;168(12):837-845.

8. West NA, Dart RC. Prescription opioid exposures and adverse outcomes among older adults. Pharmacoepidemiol Drug Saf*.* 2016;25(5):539-544.

9. Cochran G, Rosen D, McCarthy RM, Engel RJ. Risk factors for symptoms of prescription opioid misuse: do older adults differ from younger adult patients? J Gerontol Soc Work*.* 2017:1-15.

10. Park J, Lavin R. Risk factors associated with opioid medication misuse in community-dwelling older adults with chronic pain. Clin J Pain*.* 2010;26(8):647-655.

11. Gerlach LB, Olfson M, Kales HC, Maust DT. Opioids and other central nervous system-active polypharmacy in older adults in the united states. J Am Geriatr Soc. 2017.

12. Chang HY, Murimi I, Faul M, Rutkow L, Alexander GC. Impact of Florida's prescription drug monitoring program and pill mill law on high-risk patients: A comparative interrupted time series analysis. Pharmacoepidemiol Drug Saf. 2018.

13. Moyo P, Simoni-Wastila L, Griffin BA, Onukwugha E, Harrington D, Alexander GC, et al. Impact of prescription drug monitoring programs (PDMPs) on opioid utilization among Medicare beneficiaries in 10 US States. Addiction. 2017;112(10):1784-1796.

14. Wei YJ, Chen C, Schmidt SO, LoCiganic WH, Winterstein AG. Trends in prior receipt of prescription opioid or adjuvant analgesics among patients with incident opioid use disorder or opioid-related overdose from 2006 to 2016. Drug Alcohol Depend. 2019;204:107600.

15. Kuo YF, Raji MA, Chen NW, Hasan H, Goodwin JS. Trends in opioid prescriptions among Part D Medicare recipients from 2007 to 2012. Am J Med*.* 2016;129(2):221 e221-230.

16. The Centers for Medicare and Medicaid Services. Opioid Oral Morphine Milligram Equivalent (MME) Conversion Factors. In:2018. [cited 2021 January 1]. Available from: [https://www.hhs.gov/guidance/document/opioid-oral-morphine-milligram-equivalent-mme-conversion-factors-0]

17. Nagin DS, Odgers CL. Group-based trajectory modeling in clinical research. Annu Rev Clin Psychol*.* 2010;6:109-138.
