# Supplementary material for: Trajectories of prescription opioid dose and risk of opioid-related adverse events among older Medicare beneficiaries in the United States: A nested case–control study
Source: PLoS Med. 2022 Mar 15;19(3):e1003947. doi: 10.1371/journal.pmed.1003947 (PMC8923459; doi:10.1371/journal.pmed.1003947)
Supplement: S2 Text — (DOCX) [file pmed.1003947.s003.docx]

**S2 Text:** **Sensitivity analysis with a cohort study design**

**Purpose**: We conducted a sensitivity analysis using a cohort study design to test the association of prescribed opioid dose trajectories and risk for opioid-related adverse events (ORAEs) among older Medicare patients with chronic non-cancer pain (CNCP) who were new opioid users.

**Sample selection**: We used the opioid new user cohort (n=380,272) with CNCP created for this study, with the date of opioid initiation designated as the cohort entry. From this cohort, we required each patient to have at least 6 months of observations between cohort entry and exit dates (i.e., ORAE event, cancer diagnosis, receiving hospice care, death, Medicare disenrollment, or study end [12/31/2018]) to measure trajectories of prescribed opioid dose (i.e., key exposure). To include study individuals who were considered at risk for developing ORAEs, we further required patients to use opioids for at least 3 months during any 6 months identified between cohort entry and exit dates [1,2]. After applying the sample selection criteria, we obtained a cohort of 101,577 older Medicare patients with CNCP who initiated opioids and continued to use opioids for at least 3 months after the cohort entry.

In this cohort design, for each individual, we randomly selected one 6-month observation period during which opioid was used for at least 3 months between cohort entry and exit dates for measuring the key exposure—trajectories of prescribed opioid dose. This random selection approach ensures that all observed 6-month periods of opioid use within individuals have the equal chance to be chosen, thus increasing the representation of the exposure assessment in the analysis. The random selection approach has been commonly used by claims-based studies to estimate population-based estimates of drug exposure [3,4].

Here the “index date” was the day following the end of the randomly selected 6-month period to avoid immortal bias [5]. Patients were followed from the index date until an ORAE event, cancer diagnosis, receiving hospice care, death, Medicare disenrollment, study end (12/31/2018), or end of 180 days following the index date. We censored patients at the end of 180 days to ensure any ORAE that occurred during this period can be attributed to the effect of the key exposure [6].

**Exposure measurement**: Following the same approach used in the main analysis conducted by a nested case-control design, in this sensitivity analysis, we measured the prescribed opioid dose in morphine milligram equivalents (MMEs) each month over the randomly selected 6-month period for each eligible individual. We used group-based trajectory modeling (GBTM) to identify clusters of individuals, with each cluster following approximately a similar trajectory of prescribed opioid dose.

We fitted GBTM models and found a model with 4 trajectories was optimal within the recommended criteria. As shown in **S4 Fig**, these 4 groups are: gradual dose discontinuation (from <7 to 0 daily MME, 32.0%), gradual dose increase (from 0 to <7 daily MME, 12.0%), consistent low-dose (between 2 and 5 daily MME, 37.0%), and consistent high-dose group (>12 daily MME, 18.5%). These 4 trajectory groups resemble the 4 groups observed in the main analysis, despite having slightly different opioid dose ranges. The difference is mainly because of the difference in timing of exposure measurement, with the opioid dose trajectories being measured during the 6 months preceding the incident ORAE event or matched date in the main analysis, whereas trajectories being measured over a randomly selected 6-month period between cohort entry and exit dates in the sensitivity analysis.

Hereafter, we refer to the gradual dose discontinuation group as *Group 1,* gradual dose increase as *Group 2*, consistent low-dose as *Group 3*, and the consistent high-dose group as *Group 4*. We compared baseline characteristics across the four trajectory groups using the Chi-Square test for categorical variables and ANOVA test for the continuous variable (i.e., duration of opioid use since opioid initiation) (**S5 Table**).

**Measurement of covariates**: We measured the covariates that were also assessed in the main analysis at baseline, defined as 6 months between -12 to -7 months before the randomly selected 6-month period for trajectory measurement. To account for opioid exposure time, we also calculated the duration of opioid use between opioid initiation and the day before the randomly selected 6-month period for each individual.

**Statistical analysis**

We used a propensity score (PS) approach to account for differences in baseline covariates between identified trajectories of prescribed opioid groups (key exposure). Because our key exposure of interest had 4 trajectory groups, we fitted 3 separate pooled multivariable logistic regression models, with Group 2, Group 3, and Group 4 as the dependent variable, respectively, compared to Group 1 as the reference group, to estimate a summary propensity score (PS)—the likelihood of being assigned the target versus reference trajectory group conditioned on observed baseline covariates. For each pair of groups (i.e., Group 2 vs Group1, Group 3 vs Group1, and Group4 vs Group1), we then weighed the sample by inverse probability of treatment weight (IPTW). Unlike conventional covariate adjustments, IPTW adjusts for baseline confounders by assigning weights to individuals, and thus creates a pseudo sample in which all observed potential confounders are equally distributed between trajectory groups, yielding results that approximate causal relationships. In IPTW, the target group received weights of the inverse of the estimated PS, whereas the reference group received weights equal to the inverse of 1 minus the estimated PS. We truncated weights at the 1st and 99th percentiles to reduce the influence of outliers on final estimates.

We assessed covariates balance using standardized mean differences (SMDs), wherein an SMD>0.10 indicates imbalance [7]. As indicated in **S6 Table,** all baseline characteristics, except for the duration of opioid use since opioid initiation, were balanced between the target group and the reference group. To account for opioid exposure time, we further adjusted for the duration of opioid use since drug initiation as a covariate in the final weighted models.

To quantify the association between identified trajectories of prescribed opioid dose and ORAEs within 180 days of the index date, we used 3 separate IPTW weighted Cox proportional hazard models to calculate adjusted hazard ratios (HRs) and 95% confidence intervals (CIs) of time until an ORAE event in the Group 2, Group 3, and Group 4, respectively, compared to Group 1. In each model, we used Schoenfeld residuals to test the proportional hazard assumption for the key exposure of interest and detected no violation. To understand whether the risk of ORAEs varied by follow-up time, we also calculated the HRs and 95% CI of ORAEs within 30 days, 31-60 days, 61-90 days, and 91-180 days. Results of the unadjusted and adjusted association between trajectories of prescribed opioid dose and risk for ORAEs were reported in **S7 Table**.

**References**

1. Von Korff M, Walker RL, Saunders K, Shortreed SM, Thakral M, Parchman M, et al. Prevalence of prescription opioid use disorder among chronic opioid therapy patients after health plan opioid dose and risk reduction initiatives. Int J Drug Policy. 2017;46:90-8.

2. Turner JA, Saunders K, Shortreed SM, Rapp SE, Thielke S, LeResche L, et al. Chronic opioid therapy risk reduction initiative: impact on urine drug testing rates and results. J Gen Intern Med. 2014;29(2):305-11.

3. Wei YJ, Zhu Y, Liu W, Bussing R, Winterstein AG. Prevalence of and Factors Associated With Long-term Concurrent Use of Stimulants and Opioids Among Adults With Attention-Deficit/Hyperactivity Disorder. JAMA Netw Open. 2018;1(4):e181152.

4. Chen C, Lo-Ciganic WH, Winterstein AG, Tighe P, Wei YJ. Concurrent Use of Prescription Opioids and Gabapentinoids in Older Adults. Am J Prev Med. 2021; S0749-3797(21)00510-9. doi: 10.1016/j.amepre.2021.08.024. Online ahead of print.

5. Suissa S. Immortal time bias in pharmaco-epidemiology. Am J Epidemiol. 2008;167(4):492-9.

6. Langan SM, Schmidt SA, Wing K, Ehrenstein V, Nicholls SG, Filion KB, et al. The reporting of studies conducted using observational routinely collected health data statement for pharmacoepidemiology (RECORD-PE). BMJ. 2018;363:k3532.

7. Austin PC. Balance diagnostics for comparing the distribution of baseline covariates between treatment groups in propensity-score matched samples. Stat Med. 2009;28(25):3083-107.
