# Supplementary material for: Trajectories of prescription opioid dose and risk of opioid-related adverse events among older Medicare beneficiaries in the United States: A nested case–control study
Source: PLoS Med. 2022 Mar 15;19(3):e1003947. doi: 10.1371/journal.pmed.1003947 (PMC8923459; doi:10.1371/journal.pmed.1003947)
Supplement: S1 Table — (DOCX) [file pmed.1003947.s004.docx]

**S1 Table.** *ICD-9-CM or ICD-10-CM* Codes and Procedures for Disease Conditions and Service Care Considered in the Study

| **Disease, Condition, or Service Care** | ***ICD-9-CM or ICD-10-CM* codes or procedures** | **Algorithm** |
| --- | --- | --- |
| Incident opioid-related adverse events | Opioid abuse or dependence (ICD-9)  305.50 (Opioid abuse – unspecified)  305.51 (Opioid abuse – continuous)  305.52 (Opioid abuse – episodic)  304.00 (Opioid type dependence – unspecified)  304.01 (Opioid type dependence – continuous)  304.02 (Opioid type dependence – episodic)  304.70 (Combinations of opioid type drug with any other – unspecified)  304.71 (Combinations of opioid type drug with any other – continuous)  304.72 (Combinations of opioid type drug with any other – episodic) | At least 1 inpatient, SNF, HHA, or carrier claim with disease code |
|  | Opioid poisoning (ICD-9):  965.00 (Poisoning – opium (alkaloids), unspecified)  965.01 (Poisoning – heroin)  965.02 (Poisoning – methadone)  965.09 (Poisoning – opiates and related narcotics, other)  970.1 (Poisoning by opiate antagonists)  E850.0 (Accidental poisoning by heroin)  E850.1 (Accidental poisoning by methadone)  E850.2 (Accidental poisoning by other opiates and related narcotics) |  |
|  | Adverse effects of opioids (ICD-9):  E935.0 (Heroin causing adverse effects in therapeutic use),  E935.1 (methadone causing adverse effects in therapeutic use),  E935.2 (other opiates and related narcotics causing adverse effects in therapeutic use), and  E940.1 (opiate antagonists causing adverse effects in therapeutic use) |  |
|  | Corresponded ICD-10 codes for opioid abuse, opioid dependence, opioid poisoning, and adverse effects of opioids are listed at https://www.hcup-us.ahrq.gov/reports/statbriefs/sb258-Opioid-Hospitalizations-Rural-Metro-Hospitals-2016.jsp |  |
| Chronic pain |  |  |
| Musculoskeletal | 274.x, 710.x-729.x (exclude 723.4, 724.3, 724.4, 729.1, 729.2), A18.01-A18.02, A52.16, D48.1, E08.61x, E09.61x, E10.61x, E11.61x, E13.61x, M00-M02, M04.02-M04.09, M05-M19, M1A, M20.10, M21.61-M21.62, M22-M25, M32-M36, M43.2-M43.8X9, M45-M48, M49.80, M50, M51, M53, M54, M60.0-M60.2, M61-M63, M65-M67, M70-M72, M75-M77, M79, M96.1, M99.2-M99.7, N20.0, Q68.6, R25.2, R26.2, R29.8x | At least 1 inpatient, SNF, HHA, HOP, or carrier claim with disease code |
| Neuropathic | 053.1x, 249.6, 250.6, 307.89, 336.x, 337.x, 338.0, 340, 350.x, 351.x, 352.1, 353.x-355.x, 357.1, 357.2-357.4, 357.8, 357.9, 723.4, 724.3, 724.4, 729.1, 729.2,  A52.15, B02 (exclude B02.1), EXX.4, EXX.610, EXX.65 (where X in “08”-“13”), E10.4, F45.42, G13.0, G13.1, G32.0, G35, G50- G52.1, G54-G59, G61.8, G61.9, G62.8, G62.9, G63-G65, G89.0, G90.0, G90.5, G95, G99.0-G99.2, M05.5, M54.13-M54.18, M54.3, M54.4, M60.8, M60.9, M79.1, M79.2, M79.7 |  |
| Idiopathic | 338.2, 338.4, 780.96, G89, R52 |  |
| Cancer diagnosis | CCS11-CCS43 | HCUP CCS for *ICD-9-CM or ICD-10-CM* |
| Hospice care | Admission date of hospice claims | At least 1 hospice claim |
| Palliative Care | DX: V 66.7  Provide specialty code: 17 | At least 1 inpatient, SNF, HHA, HOP, carrier, or DME claim with disease code; or at least 1 inpatient, SNF, HHA, HOP with provider specialty code |
| Tobacco use | 305.1, 649.0x, 989.84,F17.x, O99.33x, T65.21xA, Z72.0 | At least 1 inpatient, SNF, HHA, HOP, carrier, or DME claim with disease code |
| Alcohol use disorders | 291.x, 303.x, 305.0x, 357.5, 425.5, 535.3x, 571.0-571.3, 760.71, 980.0, V65.42, V79.1, E860.0, F10.x (excluding F10.11, F10.13, F10.21, F10.93), G62.1, I42.6, K29.2x, K70.x, P04.3, Q86.0, T51.0XxA, Z71.4x |  |
| **Comorbidities associated with pain or pain management** |  |  |
| Mental disorder | Anxiety disorders (CCS 651), mood disorders (CCS 657), schizophrenia and other psychotic disorders (CCS 659) | HCUP CCS for *ICD-9-CM or ICD-10-CM* |
| Diabetes | Diabetes mellitus without complication (CCS 49), diabetes mellitus with complications (CCS 50) |  |
| Cardiovascular diseases | Heart valve disorders (CCS 96), coronary atherosclerosis and other heart disease (CCS 101), pulmonary heart disease (CCS 103), cardiac dysrhythmias (CCS 106), congestive heart failure; nonhypertensive (CCS 108), acute cerebrovascular disease (CCS 109), occlusion or stenosis of precerebral arteries (CCS 110), other and ill-defined cerebrovascular disease (CCS 111), peripheral and visceral atherosclerosis (CCS 114) |  |
| Hypertension | essential hypertension (CCS 98), hypertension with complications and secondary hypertension (CCS 99) |  |
| Pulmonary condition | Pneumonia (except that caused by tuberculosis or sexually transmitted disease) (CCS 122), acute bronchitis (CCS 125), other upper respiratory infections (CCS 126), chronic obstructive pulmonary disease and bronchiectasis (CCS 127), asthma (CCS 128), pleurisy; pneumothorax; pulmonary collapse (CCS 130), respiratory failure; insufficiency; arrest (CCS 131), other lower respiratory disease (CCS 133) |  |
| Kidney disease | Nephritis; nephrosis; renal sclerosis (CCS 156), acute and unspecified renal failure (CCS 157), chronic kidney disease (CCS 158), other diseases of kidney and ureters (CCS 161) |  |
| Gastrointestinal tract disorder | Gastrointestinal hemorrhage (CCS 153), other gastrointestinal disorders (CCS 155), digestive congenital anomalies (CCS 214) |  |
| Neurodegenerative disease | Parkinson disease (CCS 79), other hereditary and degenerative nervous system conditions (CCS 81) |  |
| Injuries | 800-897, 900-959, 990-999,S00-S99, T07, T14-T34, T66-T88 | At least 1 inpatient, SNF, HHA, HOP, or carrier claim with disease code |
| Infections due to non-sterile opioid injection | 042, V08, 070.41, 070.44, 070.51, 070.54, 070.7x, V0262, 035, 040.0, 569.61, 681, 682, 785.4, 728.86, 038.2, 790.7, 421, 711.0, 730.0, 730.2, 320, A40.3, A46, A48.0, B17, B18.2, B19.2, B20, E08.52, E09.52, E10.52, E11.52, E13.52, G00, G01, G042, I33, I39, I70.36, I70.46, I70.56, I70.66, I70.76, I73.01, I96, K12.2, K94.02, K94.12, L02.01, L02.11, L02.21, L02.31, L02.41, L02.51, L02.61, L02.81, L02.91, L03, L98.3, M00, M72.6, M86, R78.81, Z21 |  |

Abbreviations: SNF, skilled nursing facility; HHA, home health agency; HOP, hospital outpatient; HCUP, Healthcare Cost and Utilization Project;

CCS, Clinical Classification Software; DME, Duration Medical Equipment
