# Supplementary material for: Trajectories of prescription opioid dose and risk of opioid-related adverse events among older Medicare beneficiaries in the United States: A nested case–control study
Source: PLoS Med. 2022 Mar 15;19(3):e1003947. doi: 10.1371/journal.pmed.1003947 (PMC8923459; doi:10.1371/journal.pmed.1003947)
Supplement: S2 Table — (DOCX) [file pmed.1003947.s005.docx]

**S2 Table.** Study Prescription Opioids Approved by the US Food and Drug Administration for Use in the US Market from 2011 to 2018

| **Generic Drug Name** | **Controlled Substance Schedule** |
| --- | --- |
| Butorphanol | IV |
| Codeine | II |
| Dihydrocodeine | II |
| Fentanyl | II |
| Hydrocodone | II |
| Hydromorphone | II |
| Levorphanol | II |
| Meperidine | II |
| Methadone | II |
| Morphine | II |
| Nalbuphine | Not controlled |
| Opium | II |
| Oxycodone | II |
| Oxymorphone | II |
| Pentazocine | IV |
| Remifentanil | II |
| Sufentanil | II |
| Tramadol | IV |
