# Supplementary material for: Trajectories of prescription opioid dose and risk of opioid-related adverse events among older Medicare beneficiaries in the United States: A nested case–control study
Source: PLoS Med. 2022 Mar 15;19(3):e1003947. doi: 10.1371/journal.pmed.1003947 (PMC8923459; doi:10.1371/journal.pmed.1003947)
Supplement: S3 Table — (DOCX) [file pmed.1003947.s006.docx]

**S3 Table.** Criteria Used to Decide an Optimal Solution for Number of Latent Groups Among Case and Control Patients^a^

| **Number of groups** | **BIC** | **AIC** | **Posterior probability** | **Group size (% of total sample)** |
| --- | --- | --- | --- | --- |
| 1 | -57713.86 | -57717.44 | Group 1: 1.000 | 100 |
| 2 | -51892.28 | -51865.35 | Group 1: 0.987  Group 2: 0.976 | 66.4  33.6 |
| 3 | -50887.73 | -50847.33 | Group 1: 0.945  Group 2: 0.924  Group 3: 0.981 | 34.0  31.7  34.3 |
| 4^b^ | -50075.90 | -50011.93 | Group 1: 0.952  Group 2: 0.971  Group 3: 0.958  Group 4: 0.939 | 23.5  30.3  24.3  22.0 |
| 5^b^ | -49470.09 | -49398.30 | Group 1: 0.945  Group 2: 0.981  Group 3: 0.935  Group 4: 0.952  Group 5: 0.958 | 21.2  17.9  11.6  27.3  22.0 |

Abbreviations: AIC, Akaike Information Criteria; BIC, Bayesian Information Criteria.

^a^ Following recommended procedures, we fitted group-based trajectory models with 1 to 5 groups, and each model was tested with linear, quadratic, and cubic terms to determine the best shapes that fit the data. We then determined the optimal number of trajectory groups based on (1) BIC and AIC, with a lower BIC/AIC indicating a better model fit; (2) model adequacy, evidenced by an average posterior probability of at least 0.7 in each group identified; (3) sufficient group size that constituted at least 5% of the total sample; and (4) clinical relevance. These procedures indicated that a model with 4 trajectories was optimal.

^b^ Both models (ie, model with 4 trajectories and model with 5 trajectories) have a good model fit with relative low BIC and AIC, model adequacy (posterior probability of at least 0.7 in each group identified), and sufficient group size (all groups identified constituted at least 5% of the total sample). Clinical relevance: the model with 4 trajectories represents a valid clinical picture by identifying 4 distinct groups. The model with 5 trajectories is suboptimal because group 2 and group 3 identified in this model represent a similar group of individuals with a consistent low dose of prescribed opioids.
