# Supplementary material for: Trajectories of prescription opioid dose and risk of opioid-related adverse events among older Medicare beneficiaries in the United States: A nested case–control study
Source: PLoS Med. 2022 Mar 15;19(3):e1003947. doi: 10.1371/journal.pmed.1003947 (PMC8923459; doi:10.1371/journal.pmed.1003947)
Supplement: S4 Table — (DOCX) [file pmed.1003947.s007.docx]

**S4 Table.** Adjusted Association of Trajectories of Prescription Opioid Dose with Risk for Specific Types of Opioid-Related Adverse Events

| **Characteristics** | Opioid misuse or dependence cases (n=1,723) vs. matched controls (n=1,723) | | | | Opioid poisoning cases (n=1,399) vs. matched controls (n=1,399) | | | |
| --- | --- | --- | --- | --- | --- | --- | --- | --- |
|  | Unadjusted OR  (95% CI) | P-value | Adjusted**^2^** OR  (95% CI) | P-value | Unadjusted OR  (95% CI) | P-value | Adjusted**^2^**  OR  (95% CI) | P-value |
| **Dose trajectory group** |  |  |  |  |  |  |  |  |
| Gradual dose discontinuation group | 1.00 Reference |  | 1.00 Reference |  |  |  | 1.00 Reference |  |
| Gradual dose increase group | 1.86 (1.49-2.32) | <0.001 | 2.05 (1.61-2.61) | <0.001 | 6.09 (4.74-7.81) | <0.001 | 7.16 (5.42-9.45) | <0.001 |
| Consistent low-dose group | 4.17 (3.32-5.24) | <0.001 | 3.86 (3.00-4.99) | <0.001 | 3.17 (2.46-4.08) | <0.001 | 3.52 (2.66-4.64) | <0.001 |
| Consistent moderate-dose group | 14.35 (10.80-19.08) | <0.001 | 11.83 (8.43-16.60) | <0.001 | 4.41 (3.41-5.71) | <0.001 | 5.88 (4.23-8.16) | <0.001 |
| **Race/ethnicity** |  |  |  |  |  |  |  |  |
| White | 1.00 Reference |  | 1.00 Reference |  |  |  | 1.00 Reference |  |
| Black | 0.99 (0.78-1.21) | 0.896 | 1.06 (0.77-1.46) | 0.751 | 0.68 (0.51- 0.89) | 0.006 | 0.73 (0.51-1.04) | 0.083 |
| Other^1^ | 0.94 (0.75-1.17) | 0.569 | 1.14 (0.84-1.56) | 0.392 | 0.72 (0.56 – 0.91) | 0.006 | 0.74 (0.54-1.04) | 0.065 |
| **Low-income subsidy status**  (Yes vs no) | 1.17 (1.02-1.35) | 0.027 | 0.92 (0.75-1.13) | 0.461 | 0.92 (0.78 – 1.08) | 0.316 | 0.96 (0.76-1.20) | 0.697 |
| **Region** |  |  |  |  |  |  |  |  |
| South | 1.00 Reference |  | 1.00 Reference |  |  |  | 1.00 Reference |  |
| Northeast | 0.88 (0.71-1.08) | 0.223 | 0.98 (0.74-1.28) | 0.838 | 1.48 (1.19- 1.84) | 0.001 | 1.28 (0.98-1.67) | 0.070 |
| Midwest | 0.58 (0.48-0.70) | <0.001 | 0.55 (0.43-0.70) | <0.001 | 1.48 (1.23- 1.78) | <0.001 | 1.51 (1.21-1.90) | 0.001 |
| West | 1.09 (0.83-1.19) | 0.966 | 0.99 (0.78-1.26) | 0.920 | 1.62 (1.30 – 2.02) | <0.001 | 1.71 (1.30-2.25) | <0.001 |
| **Tobacco or Alcohol use disorder** (Yes vs no) | 2.04 (1.61-2.57) | <0.001 | 1.79 (1.32-2.44) | <0.001 | 1.40 (1.07- 1.84) | 0.014 | 1.02 (0.73-1.43) | 0.920 |
| **Chronic pain diagnosis** |  |  |  |  |  |  |  |  |
| Musculoskeletal pain (Yes vs no) | 2.21 (1.81-2.70) | <0.001 | 1.32 (1.02-1.71) | 0.032 | 1.26 (1.04 – 1.54) | 0.021 | 0.98 (0.76-1.26) | 0.855 |
| Neuropathic pain (Yes vs no) | 2.02 (1.75-2.33) | <0.001 | 1.46 (1.21-1.76) | <0.001 | 1.66 (1.42 – 1.95) | <0.001 | 1.55 (1.27-1.90) | <0.001 |
| Idiopathic pain (Yes vs no) | 3.14 (2.61-3.71) | <0.001 | 1.83 (1.44-2.34) | <0.001 | 1.71 (1.39 – 2.11) | <0.001 | 1.27 (0.98-1.65) | 0.073 |
| **Clinical conditions** |  |  |  |  |  |  |  |  |
| Mental health (Yes vs no) | 1.65 (1.42-1.91) | <0.001 | 1.29 (1.05-1.57) | 0.011 | 1.39 (1.18- 1.63) | <0.001 | 1.25 (1.02-1.55) | 0.033 |
| Diabetes (Yes vs no) | 1.02 (0.89-1.17) | 0.782 | 0.97 (0.80-1.17) | 0.743 | 1.08 (0.92- 1.26) | 0.348 | 0.82 (0.67-1.01) | 0.057 |
| Cardiovascular disease (Yes vs no) | 1.25 (1.09-1.43) | 0.001 | 0.97 (0.79-1.18) | 0.730 | 1.58 (1.35- 1.85) | <0.001 | 1.47 (1.20-1.81) | <0.001 |
| Hypertension (Yes vs no) | 1.16 (0.99-1.36) | 0.067 | 1.08 (0.85-1.36) | 0.514 | 1.12 (0.93-1.34) | 0.234 | 0.97 (0.76-1.24) | 0.807 |
| Pulmonary condition (Yes vs no) | 1.30 (1.14-1.49) | <0.001 | 1.05 (0.84-1.30) | 0.673 | 1.37 (1.17-1.59) | <0.001 | 1.15 (0.91-1.44) | 0.241 |
| Kidney disease (Yes vs no) | 1.11 (0.94-1.31) | 0.212 | 1.18 (0.94-1.48) | 0.173 | 1.48 (1.24-1.76) | <0.001 | 1.41 (1.13-1.76) | 0.003 |
| Gastrointestinal disorder (Yes vs no) | 1.33 (1.14-1.55) | 0.001 | 1.12 (0.91-1.39) | 0.285 | 1.37 (1.16-1.62) | 0.001 | 1.14 (0.91-1.42) | 0.248 |
| Respiratory infections (Yes vs no) | 1.53 (1.31-1.78) | <0.001 | 1.36 (1.07-1.73) | 0.013 | 1.26 (1.07-1.48) | 0.007 | 0.95 (0.75-1.21) | 0.687 |
| Injuries (Yes vs no) | 1.24 (1.05-1.47) | 0.011 | 1.08 (0.84-1.39) | 0.553 | 1.67 (1.39-2.00) | <0.001 | 1.07 (0.83-1.38) | 0.598 |
| Infections due to non-sterile opioid injection (Yes vs no) | 1.26 (0.99-1.60) | 0.061 | 0.88 (0.64-1.21) | 0.426 | 1.21 (0.93-1.57) | 0.149 | 0.92 (0.67-1.26) | 0.596 |
| **Medication utilization** |  |  |  |  |  |  |  |  |
| Polypharmacy (Yes vs no) | 1.80 (1.45-2.24) | <0.001 | 1.06 (0.78-1.44) | 0.696 | 1.11 (0.89-1.40) | 0.353 | 0.88 (0.65-1.19) | 0.408 |
| **Healthcare utilization** |  |  |  |  |  |  |  |  |
| Any hospital stay (Yes vs no) | 1.17 (0.99-1.40) | 0.070 | 0.75 (0.57-0.99) | 0.038 | 1.58 (1.30-1.91) | <0.001 | 1.14 (0.85-1.52) | 0.372 |
| Any ED visit (Yes vs no) | 1.17 (1.01-1.37) | 0.042 | 0.87 (0.69-1.09) | 0.210 | 1.57 (1.32-1.87) | <0.001 | 1.21 (0.96-1.52) | 0.106 |
| Any SNF stay (Yes vs no) | 1.33 (0.99-1.79) | 0.061 | 0.84 (0.54-1.30) | 0.409 | 1.60 (1.21-2.13) | <0.001 | 1.08 (0.73-1.61) | 0.676 |
| **Duration of opioid use since opioid initiation** (per 30 days) | 1.08 (1.06-1.09) | <0.001 | 1.01 (0.99-1.02) | 0.206 | 0.99 (0.98-1.01) | 0.308 | 0.98 (0.96-0.99) | <0.001 |

Abbreviations: OR, Odds Ratio; ED, emergency department; SNF, skilled nursing facility.

**^1^** Included Hispanic, Asian, Pacific Islander, and Native American individuals.

**^2^** Adjusted for race/ethnicity, low-income subsidy status, region, tobacco or alcohol use disorder, chronic pain diagnoses, clinical conditions, medication utilization, healthcare utilization, and the year of the index date.
