# Supplementary material for: Trajectories of prescription opioid dose and risk of opioid-related adverse events among older Medicare beneficiaries in the United States: A nested case–control study
Source: PLoS Med. 2022 Mar 15;19(3):e1003947. doi: 10.1371/journal.pmed.1003947 (PMC8923459; doi:10.1371/journal.pmed.1003947)
Supplement: S5 Table — (DOCX) [file pmed.1003947.s008.docx]

**S5 Table**. Sensitivity Analysis of Characteristics of Eligible Older Patients, Overall and by Defined Trajectories of Prescribed Opioid Dose, in A Cohort Design

| Characteristics ^a^ | Total sample  N=101,557 (100%) | Group 1 ^b^  n=32,517 (100%) | Group 2 ^b^  n=12,652 (100%) | Group 3 ^b^  n=37,647(100%) | Group 4 ^b^  n=18,741 (100%) | P-value across groups ^c^ |
| --- | --- | --- | --- | --- | --- | --- |
| **Age, year** |  |  |  |  |  | < 0.001 |
| Mean (SD) | 77.4 (7.8) | 76.7 (7.6) | 77.8 (7.8) | 77.3 (7.8) | 78.4 (8.3) |  |
| 65-74 | 44564 (43.9) | 15418 (47.4) | 5259 (41.6) | 16454 (43.7) | 7433 (40.0) |  |
| 75-84 | 36255 (35.7) | 11364 (35.0) | 4737 (37.4) | 13584 (36.1) | 6570 (35.0) |  |
| 85+ | 20738 (20.4) | 5735 (17.6) | 2656 (21.0) | 7609 (20.2) | 4738 (25.0) |  |
| **Female** | 70798 (69.7) | 22264 (68.5) | 8910 (70.4) | 26514 (70.4) | 13110 (70.0) | < 0.001 |
| **Race/ethnicity** |  |  |  |  |  | < 0.001 |
| White | 80674 (79.4) | 25773 (79.3) | 10124 (80.0) | 29339 (77.9) | 15438 (82.4) |  |
| Black | 9216 (9.1) | 2748 (8.5) | 1106 (8.7) | 3773 (10.0) | 1589 (8.5) |  |
| Other ^d^ | 11667 (11.5) | 3996 (12.3) | 1422 (11.2) | 4535 (12.1) | 1714 (9.2) |  |
| **LIS status** | 32324 (31.8) | 9249 (28.4) | 3902 (30.9) | 11633 (30.9) | 7536 (40.2) | < 0.001 |
| **Region** |  |  |  |  |  | < 0.001 |
| South | 43593 (42.9) | 13558 (41.7) | 1850 (14.6) | 16660 (44.3) | 7884 (42.1) |  |
| Northeast | 15318 (15.1) | 5201 (16.0) | 3103 (24.5) | 5504 (14.6) | 2763 (14.7) |  |
| Midwest | 24803 (24.4) | 7870 (24.2) | 5491 (43.4) | 8881 (23.6) | 4949 (26.4) |  |
| West | 17843(15.6) | 5888 (18.1) | 2208 (17.5) | 6602 (17.5) | 3145 (16.8) |  |
| **Tobacco or alcohol use disorder** | 8034 (7.9) | 1538 (7.8) | 999 (7.9) | 2752 (7.3) | 1745 (9.3) | < 0.001 |
| **Chronic pain diagnosis** |  |  |  |  |  |  |
| Musculoskeletal pain | 89988 (88.6) | 29315 (90.2) | 10706 (84.6) | 32968 (87.6) | 7280 (38.9) | < 0.001 |
| Neuropathic pain | 36584 (36.0) | 11442 (35.2) | 4519 (35.7) | 13343 (35.4) | 3996 (21.3) | < 0.001 |
| Idiopathic pain | 14302 (14.1) | 3986 (12.3) | 1710 (13.5) | 4610 (12.3) | 6774 (36.2) | < 0.001 |
| **Clinical conditions** |  |  |  |  |  |  |
| Mental health disorders | 28469 (28.0) | 8212(25.3) | 3555 (28.1) | 9928 (26.4) | 6774 (36.2) | < 0.001 |
| Diabetes | 43220 (42.6) | 13560 (41.7) | 5482 (43.3) | 16272 (43.2) | 7906 (42.2) | < 0.001 |
| CVD | 55724 (54.9) | 17006 (52.3) | 7096 (56.1) | 20520 (54.5) | 11102 (59.2) | < 0.001 |
| Hypertension | 79302 (78.1) | 24907 (76.6) | 9890 (78.2) | 29445 (78.2) | 15060 (80.4) | < 0.001 |
| Pulmonary condition | 54138 (53.3) | 16895 (52.0) | 6907 (54.6) | 20174 (53.6) | 10162 (54,2) | < 0.001 |
| Kidney disease | 22523 (22.2) | 6546 (20.1) | 2882 (22.8) | 8610 (22.9) | 4485 (23.9) | < 0.001 |
| Gastrointestinal disorder | 25525 (25.1) | 7604 (23.4) | 3195 (25.3) | 9239 (24.5) | 5487 (29.3) | < 0.001 |
| Respiratory infections | 25725 (25.3) | 7582(23.3) | 3327 (26.3) | 9486 (25.2) | 5330 (28.4) | < 0.001 |
| Injuries | 21386 (21.1) | 7193(22.1) | 2543 (20.1) | 7446 (19.8) | 4204 (22.4) | < 0.001 |
| Infections due to non-sterile opioid injection | 9281 (9.1) | 2728(8.4) | 1156 (9.1) | 3385 (9.0) | 2012 (10.7) | < 0.001 |
| **Polypharmacy** | 89275 (87.9) | 27911(85.8) | 10967 (88.7) | 33263 (88.4) | 17134 (91.4) | < 0.001 |
| **Healthcare utilization** |  |  |  |  |  |  |
| Any hospital stay | 21391 (21.1) | 7600(23.4) | 2353 (18.6) | 7239 (19.2) | 4199 (22.4) | < 0.001 |
| Any ED visit | 26677(26.3) | 8822 (27.1) | 3137 (24.8) | 9863 (26.2) | 4855 (25.9) | < 0.001 |
| Any SNF stay | 7472 (7.4) | 2352 (7.2) | 918 (7.3) | 2365 (6.3) | 1837 (9.8) | < 0.001 |
| **Duration of opioid use since opioid initiation, days** |  |  |  |  |  |  |
| Mean (SD) | 63.4 (138.1) | 24.2 (59.8) | 46.0 (79.8) | 46.3 (91.7) | 177.7 (243.7) | < 0.001 |

Abbreviations: SD, standard deviation; LIS, low-income subsidy; CVD, Cardiovascular diseases; ED, emergency department; SNF, skilled nursing facility

^a^ All characteristics except for the duration of opioid since opioid initiation were measured over the 6 months before the randomly selected 6-month period for exposure measurement

^b^ Group1: gradual dose discontinuation; Group2: gradual dose Increase; Group3: consistent low-Dose; Group4: consistent high-dose

^c^ Chi-Square test for categorical variables and ANOVA test for the continuous variable

^d^ Included Hispanic, Asian, Pacific Islander, and Native American individuals
