# Supplementary material for: Trajectories of prescription opioid dose and risk of opioid-related adverse events among older Medicare beneficiaries in the United States: A nested case–control study
Source: PLoS Med. 2022 Mar 15;19(3):e1003947. doi: 10.1371/journal.pmed.1003947 (PMC8923459; doi:10.1371/journal.pmed.1003947)
Supplement: S6 Table — (DOCX) [file pmed.1003947.s009.docx]

**S6 Table**. Sensitivity Analysis of Characteristics of Eligible Older Patients with Defined Trajectories of Prescribed Opioid Dose After Inverse Propensity of Treatment Weighting in A Cohort Design

| Characteristics ^a^ | Group1^b^  (n^d^=32,517) | Group2 ^b^  (n^d^=12,652 ) | SMD  (Group  2 vs 1) | Group1^b^  (n^d^=32,517 | Group3 ^b^  (n^d^=37,647) | SMD  (Group  3 vs 1) | Group1^b^  (n^d^=32,517) | Group4 ^b^  (n^d^=18,741) | SMD  (Group  4 vs 1) |
| --- | --- | --- | --- | --- | --- | --- | --- | --- | --- |
| Weighted n | 45155 (100%) | 45020 (100%) |  | 70165 (100%) | 70101 (100%) |  | 51084 (100%) | 51169 (100%) |  |
| **Age, year** |  |  |  |  |  |  |  |  |  |
| 65-74 | 20676 (45.8) | 20488 (45.5) | <0.001 | 31846 (45.5) | 31771 (45.3) | <0.001 | 22801 (44.6) | 22728 (44.4) | <0.001 |
| 75-84 | 16097 (35.7) | 16167 (35.8) |  | 24983 (35.6) | 24969 (35.6) |  | 17882 (35.0) | 17893 (35.0) |  |
| 85+ | 8383 (18.6) | 8414 (18.7) |  | 13337 (18.9) | 13361 (19.1) |  | 10401 (20.4) | 10548 (20.6) |  |
| **Female** | 31167 (69.0) | 31150 (69.2) | 0.004 | 48744 (69.4) | 48731 (69.5) | 0.002 | 35272 (69.1) | 35368 (69.1) | 0.003 |
| **Race/ethnicity** |  |  |  |  |  |  |  |  |  |
| White | 35880 (79.5) | 35735 (79.4) | <0.001 | 55072 (78.5) | 55013 (78.5) | <0.001 | 40994 (80.3) | 40972 (80.1) | <0.001 |
| Black | 3856 (8.5) | 3868 (8.6) |  | 6524 (9.3) | 6536 (9.3) |  | 4343 (8.5) | 4384 (8.6) |  |
| Other ^c^ | 5419 (12.0) | 5416 (12.0) |  | 8570 (12.2) | 8553 (12.2) |  | 5747 (11.3) | 5813 (11.4) |  |
| **LIS status** | 13154 (29.1) | 13220 (29.4) | 0.005 | 20915 (29.8) | 20919 (29.9) | 0.001 | 16601 (32.5) | 16751 (33.7) | 0.005 |
| **Region** |  |  |  |  |  |  |  |  |  |
| South | 19049 (42.2) | 19052 (42.3) | <0.001 | 30215 (43.2) | 30209 (43.1) | <0.001 | 21408 (41.9) | 21488 (42.0) | <0.001 |
| Northeast | 7051 (15.6) | 7018 (15.6) |  | 10732 (15.3) | 10706 (15.3) |  | 7934 (15.5) | 7928 (15.5) |  |
| Midwest | 10963 (24.3) | 10906 (24.2) |  | 16733 (23.8) | 16720 (23.8) |  | 12726 (24.9) | 12722 (24.9) |  |
| West | 8092 (17.9) | 8044 (17.9) |  | 12484 (17.7) | 12465 (17.8) |  | 9015 (17.7) | 9031 (17.7) |  |
| **Tobacco or alcohol use disorder** | 3532 (7.8) | 3535 (7.9) | 0.001 | 5287 (7.5) | 5274 (7.5) | <0.001 | 4261 (8.3) | 4317 (8.4) | 0.004 |
| **Chronic pain diagnosis** |  |  |  |  |  |  |  |  |  |
| Musculoskeletal pain | 49923 (88.6) | 39832 (88.5) | 0.005 | 62280 (88.8) | 62195 (88.7) | 0.002 | 46150 (90.3) | 46198 (90.3) | 0.002 |
| Neuropathic pain | 15989 (35.4) | 16125 (35.8) | 0.009 | 24838 (35.5) | 24809 (35.4) | 0.002 | 18735 (36.7) | 18972 (37.1) | 0.008 |
| Idiopathic pain | 5693 (12.6) | 5678 (12.6) | 0.001 | 8565 (12.2) | 8558 (12.2) | <0.001 | 7822 (15.3) | 7974 (15.6) | 0.008 |
| **Clinical conditions** |  |  |  |  |  |  |  |  |  |
| Mental health disorders | 11773 (26.1) | 11860 (26.3) | 0.006 | 18168 (26.0) | 18160 (25.9) | <0.001 | 14849 (29.1) | 15021 (29.4) | 0.006 |
| Diabetes | 19047 (42.2) | 19091 (42.4) | 0.005 | 29861 (42.6) | 29848 (42.6) | <0.001 | 21416 (41.9) | 21457 (41.9) | <0.001 |
| CVD | 24106 (53.4) | 24207 (53.8) | 0.008 | 37597 (53.6) | 37585 (53.6) | <0.001 | 27982 (54.8) | 28165 (55.0) | 0.005 |
| Hypertension | 34792 (77.1) | 34759 (77.2) | 0.004 | 54377 (77.5) | 54348 (77.5) | <0.001 | 39919 (78.0) | 39963 (78.1) | 0.004 |
| Pulmonary condition | 23809 (52.7) | 23887 (53.1) | 0.007 | 37105 (52.9) | 37087(52.9) | 0.001 | 27004 (52.9) | 27143 (53.0) | 0.004 |
| Kidney disease | 9433 (20.9) | 9551 (21.2) | 0.008 | 15187 (21.6) | 15206 (21.7) | 0.002 | 11037 (21.6) | 11183 (21.9) | 0.006 |
| Gastrointestinal disorder | 10801 (23.9) | 10847(24.1) | 0.004 | 16866 (24.0) | 16865 (24.1) | 0.001 | 13007 (25.5) | 13149 (25.7) | 0.005 |
| Respiratory infections | 10908 (24.2) | 10982 (24.4) | 0.006 | 17086 (24.4) | 17093 (24.4) | <0.001 | 12873 (25.2) | 13020 (25.4) | 0.006 |
| Injuries | 9730 (21.6) | 9636 (21.2) | 0.004 | 14637 (20.8) | 14595 (20.8) | <0.001 | 11389 (22.3) | 11416 (22.3) | 0.004 |
| Infections due to non-sterile opioid injection | 3883 (8.6) | 3906 (8.7) | 0.003 | 6148 (8.8) | 6145 (8.8) | <0.001 | 4727 (9.3) | 4805 (9.4) | 0.005 |
| **Polypharmacy** | 38882 (86.1) | 38897 (86.4) | 0.009 | 61194 (87.2) | 61183 (87.3) | 0.003 | 44873 (87.8) | 45081 (88.1) | 0.008 |
| **Healthcare utilization** |  |  |  |  |  |  |  |  |  |
| Any hospital stay | 9947 (22.0) | 9737 (21.6) | 0.009 | 14797 (21.1) | 14725 (21.0) | 0.005 | 11841 (23.2) | 11957 (23.4) | 0.005 |
| Any ED visit | 11974 (26.5) | 12013 (26.7) | 0.004 | 18726 (26.7) | 18699 (26.6) | <0.001 | 13685 (26.8) | 13686 (26.8) | 0.001 |
| Any SNF stay | 3260 (7.2) | 3207 (7.1) | 0.004 | 4711 (6.7) | 4697 (6.7) | 0.002 | 4189 (8.2) | 4142 (8.3) | 0.003 |
| **Duration of opioid use since opioid initiation, days** |  |  |  |  |  |  |  |  |  |
| Mean (SD) | 25 (72) | 45.0 (147) | 0.174 | 35 (121) | 37 (103) | 0.022 | 25 (79) | 168 (389) | 0.507 |

Abbreviations: SMD, standardized mean difference; LIS, low-income subsidy; CVD, cardiovascular diseases; ED, emergency department; SNF, skilled nursing facility; SD, standard deviation

^a^ All characteristics except for the duration of opioid since opioid initiation were measured over the 6 months before the randomly selected 6-month period for exposure measurement.

^b^ Group1: gradual dose discontinuation; Group2: gradual dose Increase; Group3: consistent low-Dose; Group4: consistent high-dose

^c^ Included Hispanic, Asian, Pacific Islander, and Native American individuals

^d^ Unweighted sample size
