# Supplementary material for: Trajectories of prescription opioid dose and risk of opioid-related adverse events among older Medicare beneficiaries in the United States: A nested case–control study
Source: PLoS Med. 2022 Mar 15;19(3):e1003947. doi: 10.1371/journal.pmed.1003947 (PMC8923459; doi:10.1371/journal.pmed.1003947)
Supplement: S7 Table — ORAE, opioid-related adverse event. (DOCX) [file pmed.1003947.s010.docx]

**S7 Table**. Sensitivity Analysis of Cohort Study for the Association of Defined Prescribed Opioid Dose Trajectories with Risk for Opioid-related Adverse Events

| **Opioid dose trajectory group** | **Risk for opioid-related adverse events** | | | | | | | | | |
| --- | --- | --- | --- | --- | --- | --- | --- | --- | --- | --- |
|  | **Within 180-day follow-up** | | **1-30 days** | | **31-60 days** | | **61-90 days** | | **91-180 days** | |
|  | **Unadjusted HR (95% CI)** | **Adjusted ^b^**  **HR (95% CI)** | **Unadjusted HR (95% CI)** | **Adjusted ^b^**  **HR (95% CI)** | **Unadjusted HR (95% CI)** | **Adjusted ^b^**  **HR (95% CI)** | **Unadjusted HR (95% CI)** | **Adjusted ^b^**  **HR (95% CI)** | **Unadjusted HR (95% CI)** | **Adjusted ^b^**  **HR (95% CI)** |
| **Group2 ^a^ vs Group1^a^** | 4.62  (3.80 – 5.63) | 4.39  (3.80 – 5.08) | 9.08  (6.55 – 12.58) | 8.95  (6.96 – 11.52) | 4.49  (2.50 – 8.07) | 4.45  (2.89 – 6.86) | 2.07  (1.23 – 3.50) | 1.90  (1.33 -2.71) | 2.53  (1.75- 3.68) | 2.22  (1.71-2.88) |
|  | P <0.001 | P <0.001 | P <0.001 | P <0.001 | P <0.001 | P <0.001 | P <0.001 | P <0.001 | P <0.001 | P <0.001 |
| **Group3 ^a^ vs Group1^a^** | 2.21  (1.75 – 2.53) | 1.86  (1.64 – 2.11) | 3.20  (2.32 – 4.42) | 2.66  (2.15 -3.30) | 2.96  (1.77 – 4.96) | 2.85  (1.99 – 4.08) | 1.09  (0.70 -1.38) | 1.01  (0.74 -1.37) | 1.67  (1.23 -2.27) | 1.49  (1.21-1.84) |
|  | P <0.001 | P <0.001 | P <0.001 | P <0.001 | P <0.001 | P <0.001 | P=0.694 | P=0.975 | P <0.001 | P <0.001 |
| **Group4 ^a^ vs Group1^a^** | 6.26  (5.25 – 7.46) | 5.67  (4.96 – 6.48) | 7.80  (5.68 – 10.71) | 5.67  (4.96 -6.48) | 8.36  (5.08 – 13.77) | 7.46  (5.15 -10.82) | 4.46  (3.16 -5.48) | 4.76  (3.53 -6.42) | 5.39  (4.04 – 7.19) | 4.66  (3.73- 5.82) |
|  | P <0.001 | P <0.001 | P <0.001 | P <0.001 | P <0.001 | P <0.001 | P <0.001 | P <0.001 | P <0.001 | P <0.001 |

Abbreviations: HR, hazard ratio; CI, confidence interval

^a^ Group1: gradual dose discontinuation (reference); group2: Gradual dose Increase; Group3: consistent low-Dose; Group4: consistent high-dose

^b^ Cox hazard regression model with the inverse probability of weighting treatment (IPTW) to account for baseline characteristics (including categorical age, sex, race/ethnicity, low-income subsidy status, region, tobacco or alcohol use disorder, types of chronic pain, chronic conditions, polypharmacy, any hospitalization, any emergency department visit, and any skilled nursing facility service). Duration of opioid use (in days) since opioid initiation, which remained unbalanced between groups after IPTW, was adjusted as a covariate in the final IPTW weighted Cox model.
