# Supplementary figures and images for: Trajectories of prescription opioid dose and risk of opioid-related adverse events among older Medicare beneficiaries in the United States: A nested case–control study
Source: PLoS Med. 2022 Mar 15;19(3):e1003947. doi: 10.1371/journal.pmed.1003947 (PMC8923459; doi:10.1371/journal.pmed.1003947)

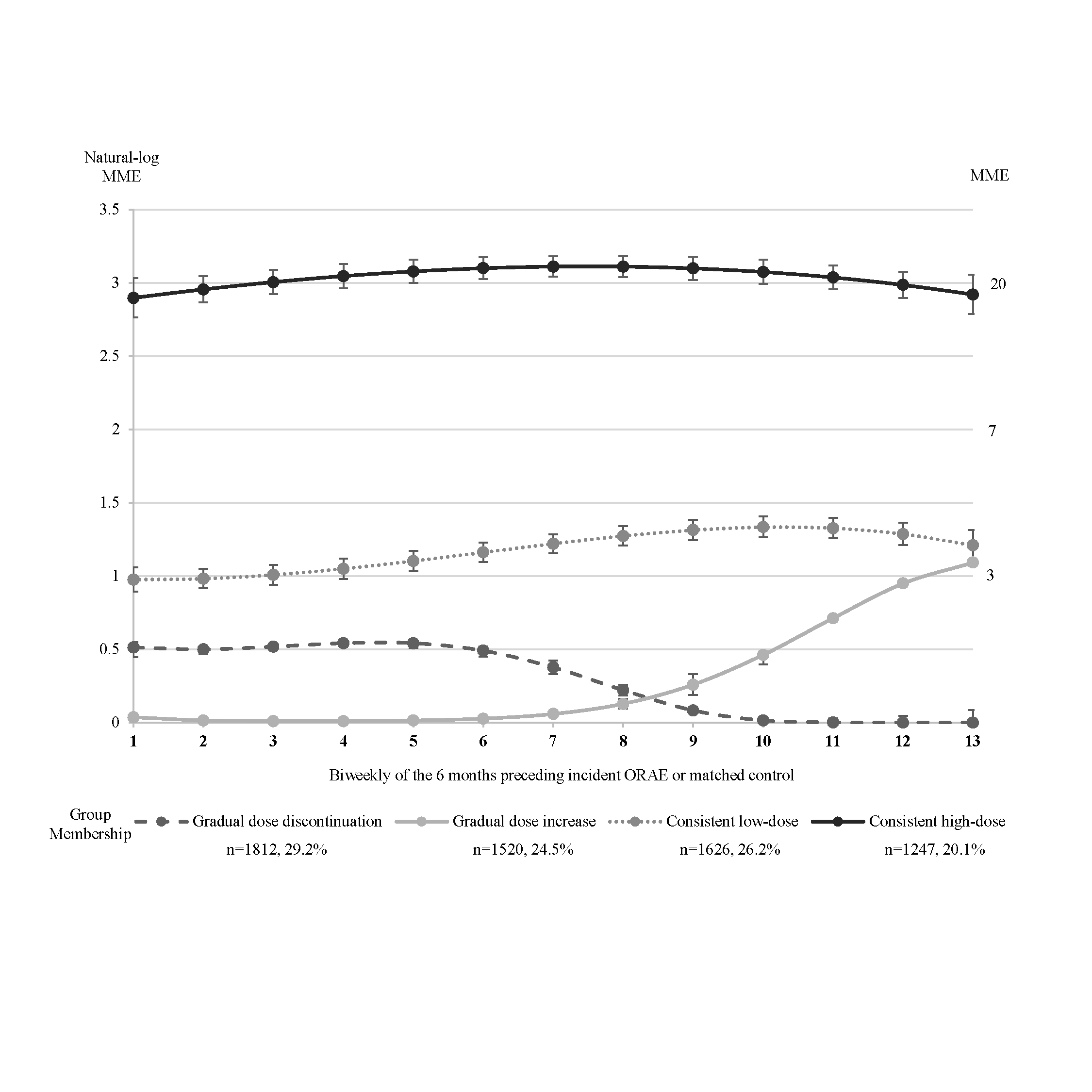

Supplement: S1 Fig — MME, morphine milligram equivalent; ORAE, opioid-related adverse event. (TIFF) [file pmed.1003947.s011.tiff]

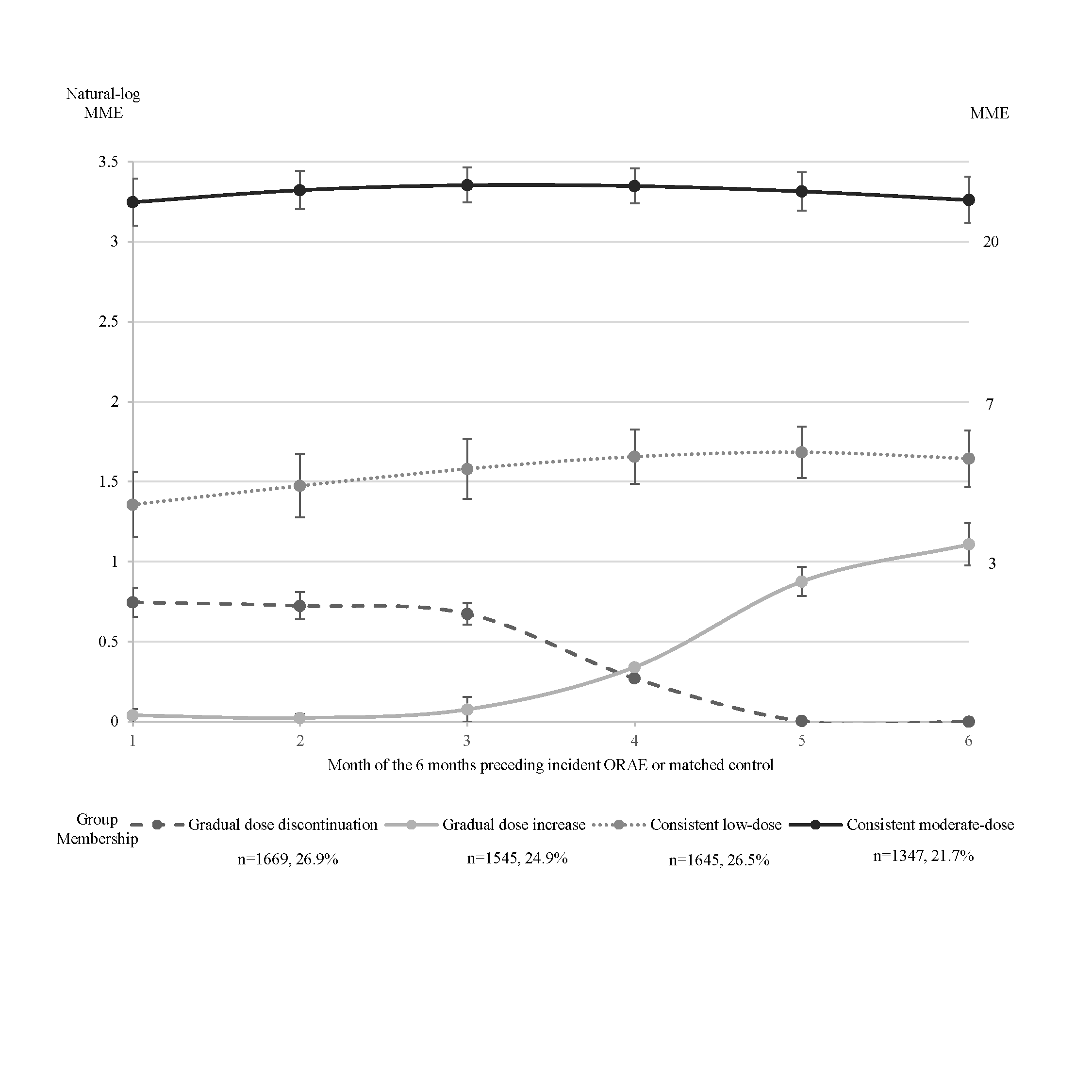

Supplement: S2 Fig — MME, morphine milligram equivalent; ORAE, opioid-related adverse event. (TIFF) [file pmed.1003947.s012.tiff]

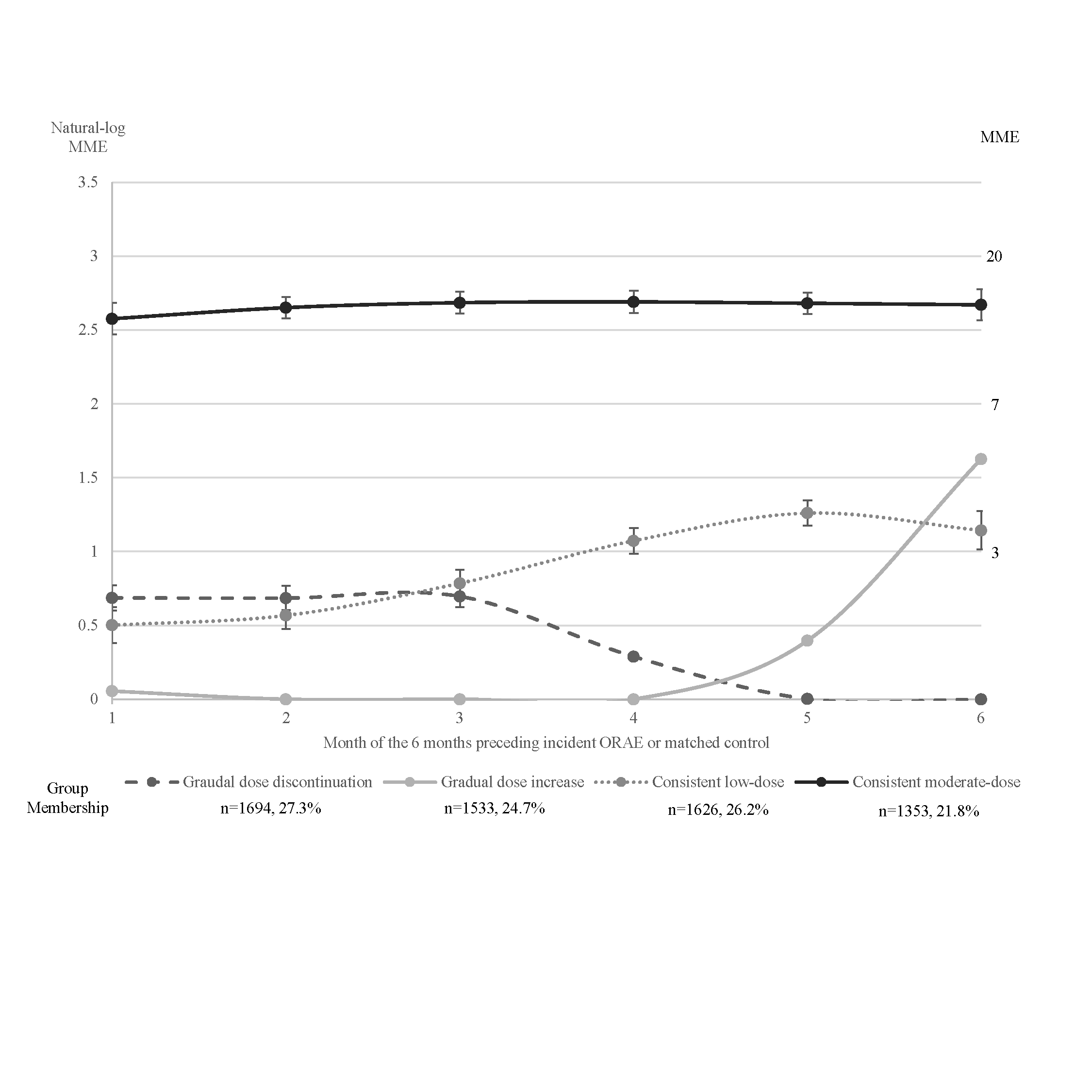

Supplement: S3 Fig — MME, morphine milligram equivalent; ORAE, opioid-related adverse event. (TIFF) [file pmed.1003947.s013.tiff]

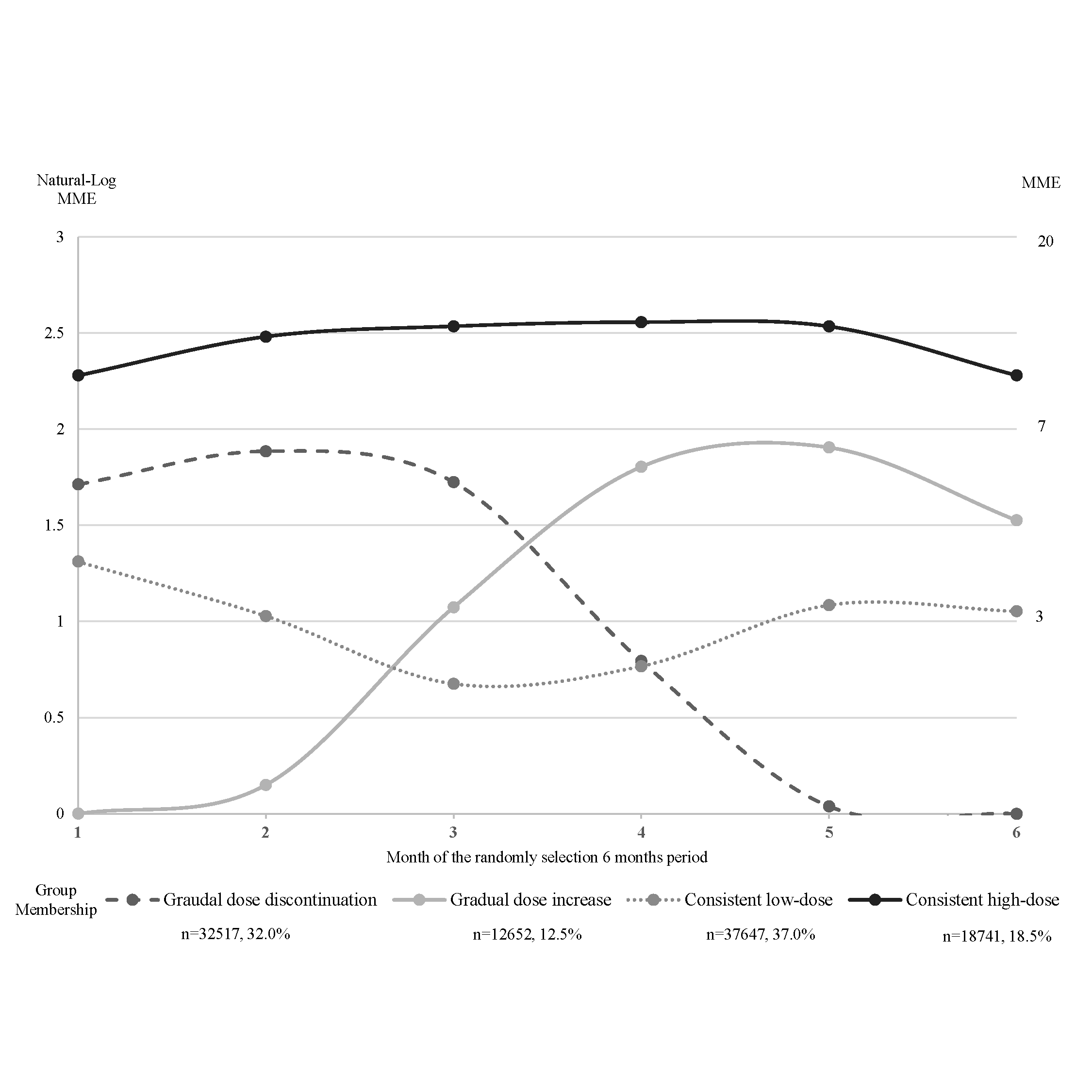

Supplement: S4 Fig — MME, morphine milligram equivalent. (TIFF) [file pmed.1003947.s014.tiff]
